# Spatiotemporal trends of neglected tropical disease hospitalizations in Ecuador over 25-years from 2000 to 2024

**DOI:** 10.1371/journal.pntd.0013688

**Published:** 2026-05-18

**Authors:** Cristina Aldaz-Barreno, Natalia Romero-Sandoval, Monsermín Gualán, Pablo Álvarez, Daniel Zurita-Loma, Gabriela Dávila Rosero, Miguel Martin, Philip J. Cooper, on behalf of the Unit on the Social and Environmental Determinants of Health Inequalities (SEDHI)

**Affiliations:** 1 School of Medicine, Universidad Internacional del Ecuador UIDE, Quito, Ecuador; 2 Grups de Recerca d’Amèrica i Àfrica Llatines – GRAAL, Barcelona, Spain; 3 Facultat de Medicina, Universitat Autonoma de Barcelona, Barcelona, Spain; 4 School of Health and Medical Sciences, City St. George’s University of London, London, United Kingdom; Universidad de Antioquía: Universidad de Antioquia, COLOMBIA

## Abstract

**Background:**

The World Health Organization identifies 21 neglected tropical diseases (NTDs) affecting millions globally. While their population burden is well recognised, less is known about the long-term trends in severe NTD-related morbidity requiring hospitalization. We analysed national trends and geographic patterns of hospitalizations attributed to NTDs in Ecuador between 2000 and 2024.

**Methods:**

We analysed hospital discharge data from Ecuador’s national registry over a 25-year period. Age-standardized hospitalization rates were estimated, and temporal trends were assessed using Joinpoint regression for the 5 most frequent NTDs. Standardized morbidity ratios (SMRs) were estimated for these NTDs for census years (2001, 2010 and 2022) to explore relative changes in hospitalization rates over time and by geography.

**Results:**

A total of 179,439 hospital discharges were attributed to NTDs, representing 0.7% of all hospitalizations. The five most frequent NTDs accounted for 97.1% of hospitalizations: dengue and chikungunya (62.4%), snakebite envenoming (20.1%), soil-transmitted helminthiases (8.7%), taeniasis and cysticercosis (3.9%), and scabies and other ectoparasitoses (2.0%). Only 0.4% of hospitalizations resulted in death. Hospitalizations were more frequent in males (54%) and younger populations (median 19 years, Q_1_ 9 – Q_3_ 37). Overall hospitalizations rates increased over time, driven primarily by arboviral infections, while non-arboviral NTDs showed declining trends: snakebite (from 2014, annual percent change -7.81%, 95% CI -11.27, -5.69, P = 0.006), soil-transmitted helminths (from 2000, -5.62%, 95% CI -6.56, -4.68, P < 0.001), and taeniasis and cysticercosis (from 2003, -10.42%, 95% CI -14.50, -9.68, P = 0.002). Relative morbidity caused by NTDs was consistently greater in Coastal and Amazon provinces, with shifts over time across regions, although taeniasis and cysticercosis morbidity remained greatest in southern Andean Provinces.

**Conclusion:**

Over 25 years in Ecuador, hospitalizations attributed to several non-arboviral NTDs declined, while arboviral infections increasingly contributed to severe NTD-related morbidity. These findings reflect trends in the most severe clinical manifestations requiring inpatient care and highlight persistent geographic inequalities, particularly in the Amazon region. Strengthening surveillance, prevention, and equitable access to timely diagnosis and care will be essential to reduce severe NTD morbidity and support progress towards national and global NTD elimination and control targets.

## Author summary

Neglected tropical diseases (NTDs) are a group of diseases affecting millions worldwide, especially in poor and marginalized populations in tropical and subtropical regions of low and middle-income countries. This study analysed hospital records over a 25-year period (2000–2024) in Ecuador—a country where 40% of the population lives in poverty, where there is considerable geoclimatic diversity but where 87% of territory has a tropical or subtropical climate—to determine how hospitalisations due to NTDs have changed over this period and which regions of the country are most affected. Most hospitalisations (97%) were caused by five diseases: the arbovirus infections, dengue and chikungunya; snakebite; intestinal worms; pork tapeworm infections; and scabies. Children and adolescents and those living in the Amazon region suffered most hospitalizations caused by NTDs, which may reflect differences in health-seeking behaviour, admission practices, and diagnostic capacity. Over time, hospitalisations from most NTDs declined although those caused by dengue and chikungunya increased. These findings highlight an urgent need to strengthen health education, disease prevention, surveillance, and access to timely diagnosis and treatment tailored to each of these NTDs, especially in the Amazon region of the country. These data help inform the country’s progress meeting its targets for NTD control and elimination by 2030 as outlined in Sustainable Development Goal 3.3.

## Introduction

Neglected Tropical Diseases (NTDs) include a diverse group of 21 conditions caused by parasites, bacteria, viruses, fungi, and non-communicable agents [1]. NTDs were estimated to affect 1.5 billion across 149 countries in 2019 where they caused 150,000 deaths and 19 million DALYs [2]. NTDs are most prevalent in poor and marginalized populations living in tropical and subtropical regions of low and middle-income countries (LMIC) and impose a substantial economic burden on LMIC economies, costing billions of dollars in health costs annually [3,4].

Optimal interventions against NTDs are addressed under Target 3.3 of the United Nations Sustainable Development Goals (SDGs). WHO’s *NTD Roadmap 2021–2030* sets a target of reducing by 90% the global population requiring interventions for NTDs by 2030 [1]. Using 2021 as the baseline year, the WHO outlined several strategic goals for NTDs by 2030: control measures for nine, elimination as a public health problem for six, interruption of transmission for three, and complete eradication for the remaining three [5]. An estimated 51 million people in Latin America required NTDs-related interventions in 2022 [6].

Hospitalizations due to NTDs represent the most severe end of the clinical spectrum and reflect health system burden rather than overall disease incidence or transmission [7–10]. This information serves as a complementary indicator to surveillance and community-level prevalence data. High rates of NTD hospitalizations place a significant economic strain on impoverished families and communities, further exacerbating preexisting vulnerabilities relating to poverty, limited access to healthcare, and discrimination based on ethnicity, language, or culture [11].

Ecuador, an upper-middle-income country of 17 million on the Pacific coast of South America, experienced an average annual population growth rate of about 1.5% between 2000 and 2024. The national health system operates under a universal health coverage framework, with a primary healthcare-oriented approach and mixed public and private financing [12]. Despite an increase in the absolute number of hospital beds, the density declined from approximately 1.55 to 1.31 beds per 1,000 population (or by 15%) between 2004 and 2023 [13]. Tropical and subtropical areas, that account for approximately 87% of the country’s land area [14], provide favourable conditions for the survival of disease vectors and the transmission of infectious pathogens. The national epidemiological surveillance system currently monitors seven notifiable NTDs [15] including rabies, dengue and chikungunya, Chagas disease, snakebite envenoming, leishmaniasis, leprosy, and onchocerciasis. Significant progress has been made in Ecuador to meet SDG 3.3 for some NTDs - the formal certification of the interruption of transmission of onchocerciasis was completed in 2015 [16] and is pending for yaws [17,18] - while for others, there are more limited published data at a national level on current status [19], or temporal trends or geographic distribution within the country (for example, myiasis [20], dengue [21–24], cysticercosis [25], and Chagas [22,23,26]). Most published data on NTDs have been limited to studies in geographically restricted populations in areas known to be highly endemic for these diseases [18,20,26,27].

The objective of the present study was to characterize long-term spatiotemporal trends in hospitalizations attributed to NTDs in Ecuador between 2000 and 2024, and to explore temporal changes and geographic heterogeneity in severe NTD-related morbidity to inform public health strategies for the management of severe NTD morbidity and contribute to assessing Ecuador’s progress towards SDG target 3.3.

## Methods

### Ethical considerations

This study used anonymized publicly accessible administrative health data from the National Institute of Statistics and Census (INEC). It did not involve contact with human participants or the use of identifiable information. In accordance with national regulations governing the use of secondary, de-identified data, formal ethical approval was not required, and the study was exempt from ethical review.

### Study design

We conducted an ecological study to analyse time trends and spatial patterns of hospitalizations due to NTDs in Ecuador from 2000 to 2024.

### Setting and selection of ICD codes

Ecuador is an upper-middle income country with high levels of inequality and where approximately 40% of the population are classified as living in poverty [28]. The country has a population of approximately 17 million and has considerable geoclimatic diversity. Ecuador lies on the equator and is bisected north-south by the Andes, dividing the country into 4 distinct geoclimatic regions (Coast, Andes, Amazon, and the Galápagos archipelago [Insular]). The country experiences wet and dry seasons that vary by month and intensity between geoclimatic regions.

Ecuador is divided administratively into 24 provinces, grouped into the four geoclimatic regions: six in the Coastal region, ten in the Andean region, five in the Amazon region, and one representing the Galápagos archipelago. The national health system operates under an integrated network model based around primary health care. All inpatient healthcare facilities—public and private—are required to report hospital discharges to the National Institute of Statistics and Censuses (INEC) using ICD-10 codes, based on the primary diagnosis recorded by the healthcare provider. INEC compiles these data through a standardized reporting system and is responsible for data storage, dissemination, and quality control. Routine data processing includes consistency checks, validation of diagnostic codes, and standardization of variables prior to release for administrative and research use [13].

### Variables and source of hospitalization data

The 21 NTDs defined by WHO are: Buruli ulcer; Chagas disease; dengue and chikungunya (dengue/chik); dracunculiasis; echinococcosis; foodborne trematodiases; human African trypanosomiasis; leishmaniasis; leprosy; lymphatic filariasis; mycetoma, chromoblastomycosis and other deep mycoses; onchocerciasis; rabies; scabies and other ectoparasitoses; schistosomiasis; soil-transmitted helminthiases; snakebite envenoming; taeniasis/cysticercosis; trachoma; and yaws. Noma was added to the NTD list in 2023 [29]. For this analysis, NTDs (S1 Table) were divided into two groups: i) the first included 14 NTDs (referred here to as endemic NTDs) considered endemic or with a known historical or persistent presence in Ecuador, characterized either by evidence of sustained local transmission or by stable environmental and ecological conditions supporting ongoing exposure risk, and included: dengue/chik; snakebite envenoming (snakebite); soil-transmitted helminths (STH); taeniasis and cysticercosis (taeniasis/cysticercosis); scabies and other ectoparasitoses (ectoparasitoses); and less frequent NTDs (named, Other NTDs)— including Chagas disease, echinococcosis, leishmaniasis, leprosy, onchocerciasis, yaws, foodborne trematodiases, mycetoma, chromoblastomycosis and other deep mycoses, and rabies; and ii) the second group (referred to as non-endemic NTDs) were those included in the WHO neglected tropical disease portfolio [29] but without evidence of local transmission (or presence) in Ecuador, and included Buruli ulcer, drancunculiasis, human African trypanosomiasis, lymphatic filariasis, noma, schistosomiasis, and trachoma. ICD-10 codes corresponding to NTDs (S1 Table) were used to identify relevant hospital discharge diagnoses from 2000 to 2024 using the national hospital discharge registry [30], and which provides aggregated monthly and annual data at provincial level. This registry provides also nominal data on sex, age, province of residence, health status at discharge (alive or dead), duration of hospitalization, ethnicity (from 2014), and area (urban/rural) of usual residence (from 2015). Dengue was classified under codes A90–A92 for the entire study period, with A97 added in 2019 following WHO recommendations [31]. ICD-10 coding for dengue was progressively aligned with the WHO 2009 dengue classification from approximately 2016, without introduction of new diagnostic categories (codes A90 and A91 remained unchanged). To minimize potential bias arising from temporal variability in coding practices, all dengue-related discharge diagnoses were aggregated into a single composite outcome. Temporal trends were additionally assessed for discontinuities suggestive of coding artefacts, particularly around the period of classification alignment, and no structural breaks were identified. Endemic NTDs were analysed at national and provincial levels. For non-endemic NTDs, area of usual residence was categorised into 3 relevant geographic areas: Ecuador, South America (outside Ecuador), and Africa.

### Statistical analysis

Descriptive statistics (absolute and relative frequencies) were calculated for the endemic NTDs (dengue/chik, snakebite, STH, taeniasis/cysticercosis, ectoparasitoses, and Other NTDs) and stratified by demographic variables. Age-standardized rates (ASR) and 95% confidence intervals (95% CI) were calculated per 100,000 population for each endemic NTD by the direct method based using the WHO world standard population [32]. Trends in NTD hospitalizations over the study period were assessed using Joinpoint regression models [33]. This method identifies statistically significant changes in temporal trends by fitting a series of log-linear regression segments joined at inflection points (“joinpoints”). Models were fitted to the natural logarithm of age-standardized rates. The analysis began with a model assuming no joinpoints (i.e., a single linear trend) and sequentially tested whether additional joinpoints significantly improved model fit. The maximum number of joinpoints was specified a priori (up to three), and the optimal number and location of joinpoints were selected using permutation tests with a significance level of 0.05. For each segment, the Annual Percentage Change (APC) and corresponding 95% confidence intervals were estimated, and statistical significance was determined by testing whether the APC differed from zero. The Average Annual Percentage Change (AAPC) was calculated as a weighted average of the segment-specific APCs over the full study period. In models with no joinpoints, the APC is constant and equivalent to the AAPC. Joinpoint regression analyses were conducted for the five most prevalent NTDs. A seasonality index was calculated for dengue/chik and snakebite using monthly hospitalization data. For each five-year interval, the mean number of hospitalizations for each calendar month was calculated across the years within that interval and divided by the overall mean monthly count for the same interval. Thus, a value of 1 indicates that the monthly frequency equals the average, values >1 indicate above-average activity, and values <1 indicate below-average activity, as previously described [34,35]. This approach allows comparison of seasonal patterns across time while accounting for changes in overall hospitalization levels. We estimated crude hospitalization rates stratified by sex and age group for each province for 2000 and 2024 using population projections [36]. To compare hospitalization rates across provinces, we calculated standardized morbidity ratios (SMRs) and their 95% confidence intervals for the 3 census years (2000, 2010, 2022) using indirect standardization, representing the ratio of observed to expected cases in each province, based on rates from the national census population, adjusted for age and sex. [37]. SMR maps were created in R version 4.5.0 using the *sf*, *rnaturalearth*, *ggplot2*, and *ggspatial* packages, with Natural Earth used as the base map source (public domain). Fixed interval scales were applied across NTD groups and census years to facilitate visual comparison of spatial patterns over time. Non-endemic NTDs were summarized using absolute frequencies per year and by origin (i.e., Ecuador, outside Ecuador but in South America, and Africa) from 2015 when data became available. Analyses were done using SPSS (V29.0.1.0), R version 4.5.0, and Joinpoint Regression Program (V5.0.2).

## Results

Between 2000 and 2024 a total of 179,439 hospitalizations, representing 0.7% of all hospitalizations, were attributed to NTDs through statutory reporting to the INEC. Table 1 summarizes national frequencies of hospital discharges for endemic NTDs, stratified by key sociodemographic variables. These include ethnicity (from 2014), length of hospital stay, health status at discharge, and urban–rural residence (from 2015). Arboviral infections (dengue/chik) were the most common, representing 62.4% of NTD hospitalizations, followed by snakebite (20.1%), STH (8.7%), taeniasis/cysticercosis (3.9%), and ectoparasitoses (2.0%). The other nine non-endemic NTDs each contributed <1%, together accounting for just 2.1% of cases.

**Table 1:** Sociodemographic characteristics and hospital stay characteristics of hospital discharges of all 21 neglected tropical diseases (NTDs) and the 14 endemic NTDs in Ecuador between 2000 and 2024.

	Total (%)	Sex		Age (years)	Health status at discharge		Hospital stay (days)	Area of residence (n = 74,880)		Minorities (n = 84,849)
		Men n (%)	Women n (%)	Median (Q1-Q3)	Alive n (%)	Dead n (%)	Median (Q1-Q3)	Urban n (%)	Rural n (%)	n (%)
All NTDs	179439 (100)	96664 (53.9)	82775 (46.1)	19 (9-37)	178632 (99.6)	807 (0.4)	3 (2-5)	61930 (82.7)	12950 (17.3)	7231 (8.5)
Endemic NTDs										
Dengue and chikungunya	111946 (62.4)	57126 (51)	54820 (49)	16 (9-31)	111570 (99.7)	376 (0.3)	3 (2-5)	47167 (88.3)	6262 (11.7)	2632 (4.4)
Snakebite envenoming	36070 (20.1)	23354 (64.7)	12716 (35.3)	28 (15-46)	35906 (99.5)	164 (0.5)	3 (2-5)	7515 (61)	4807 (39)	3332 (23.4)
Soil-transmitted helminthiases	15647 (8.7)	7450 (47.6)	8197 (52.4)	10 (4-28)	15619 (99.8)	28 (0.2)	3 (2-4)	3445 (75.9)	1096 (24.1)	975 (18.9)
Taeniasis and cysticercosis	6986 (3.9)	3595 (51.5)	3391 (48.5)	42 (28-58)	6906 (98.9)	80 (1.1)	4 (3-8)	1084 (82.7)	226 (17.3)	56 (3.7)
Scabies and other ectoparasitoses	3569 (2)	2027 (56.8)	1542 (43.2)	13 (4-57)	3526 (98.8)	43 (1.2)	5 (2-8)	1471 (83.6)	289 (16.4)	122 (6.3)
Leishmaniasis	1145 (0.6)	852 (74.4)	293 (25.6)	26 (19-48)	1142 (99.7)	3 (0.3)	4 (1-9)	253 (71.5)	101 (28.5)	54 (13.9)
Leprosy	1019 (0.6)	718 (70.5)	301 (29.5)	55 (43-68)	980 (96.2)	39 (3.8)	25 (9-92)	42 (77.8)	12 (22.2)	5 (6.5)
Mycoses	628 (0.3)	344 (54.8)	284 (45.2)	35 (16-52)	612 (97.5)	16 (2.5)	2 (1-9)	321 (87.7)	45 (12.3)	16 (3.7)
Chagas disease	494 (0.3)	309 (62.6)	185 (37.4)	62.5 (38-77)	461 (93.3)	33 (6.7)	5 (2-10)	144 (85.2)	25 (14.8)	16 (8.2)
Echinococcosis	253 (0.1)	101 (39.9)	152 (60.1)	40 (23-60)	251 (99.2)	2 (0.8)	5 (3-11)	108 (76.1)	34 (23.9)	6 (3.9)
Yaws	230 (0.1)	111 (48.3)	119 (51.7)	31 (15-52)	229 (99.6)	1 (0.4)	1 (1-3)	104 (90.4)	11 (9.6)	5 (3.8)
Foodborne trematodiases	154 (0.1)	76 (49.4)	78 (50.6)	25 (12-41)	152 (98.7)	2 (1.3)	5 (2-8)	28 (77.8)	8 (22.2)	6 (14.3)
Rabies	83 (0)	19 (22.9)	64 (77.1)	26 (20-35)	76 (91.6)	7 (8.4)	2 (1-3)	2 (100)	0 (0)	0 (0)
Onchocerciasis	8 (0)	2 (25)	6 (75)	19 (7-55.5)	8 (100)	0 (0)	2 (1.5-6)	1 (100)	0 (0)	0 (0)

NTD hospitalizations tended to be more common in males (53.9%), particularly for snakebite (64.7% of hospitalizations). In-hospital case fatality rate was low – only 0.4% of NTD hospitalizations resulted in the death of the patient although this was much higher for Chagas disease (6.7%). NTD hospitalizations were much more frequent for populations whose area of habitual residence was urban (82.7% of hospitalizations) and was affected by the strong urban bias for arbovirus infections (88.3% of hospitalizations). Only 8.5% of hospitalizations were reported for ethnic minorities (i.e., Indigenous, Montubio, and Afro-Ecuadorian), much lower than the population proportion in the 2022 national census (20.2%) [28].

Data are based on 179,439 hospital discharges attributed to NTDs between 2000 and 2024, unless otherwise specified. Values are presented as counts (percentages) or medians (interquartile range). Data availability varies by variable: area of residence was available for 2015–2024 (n = 74,880), and ethnicity (minority status: Indigenous, Afro-Ecuadorian, and Montubio) for 2014–2024 (n = 84,849). Percentages for these variables are calculated using the available denominators. All NTDs include the 21 diseases defined by WHO; endemic NTDs include those with evidence of local transmission or persistent presence in Ecuador. Mycoses – Mycetoma, chromoblastomycosis and other deep mycoses.

**Fig 1:**
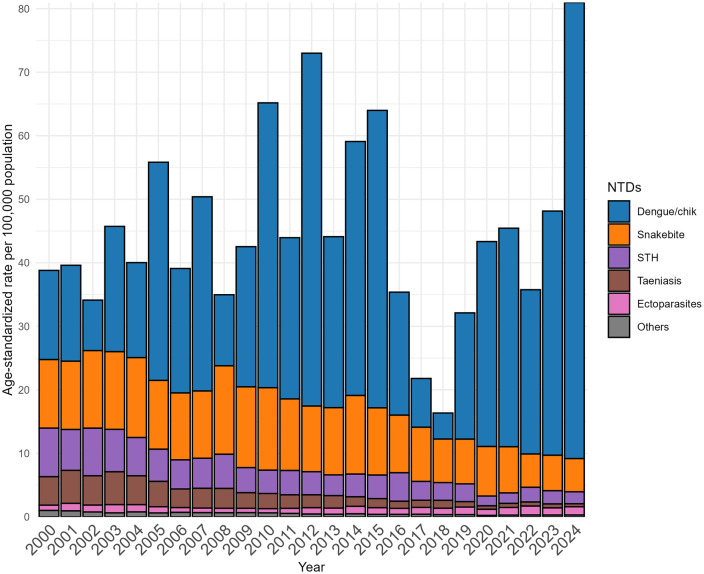
Temporal trends in age-standardized hospitalization rates for endemic neglected tropical diseases (NTDs) in Ecuador, 2000–2024. Annual age-standardized hospitalization rates (per 100,000 population) are shown for major endemic NTD groups. Rates were calculated using the direct method based on the WHO standard population. The figure illustrates the relative contribution of arboviral and non-arboviral NTDs to overall hospitalization burden over time. Dengue and chikungunya, Snakebite envenoming, soil-transmitted helminths, Taeniasis and cysticercosis, Scabies and other ectoparasitoses, and Other NTDs—including Chagas disease, echinococcosis, leishmaniasis, leprosy, onchocerciasis, yaws, foodborne trematodiases, mycetoma, chromoblastomycosis and other deep mycoses, and rabies.

Cumulative annual age-standardized rates for endemic NTDs in Ecuador are shown in Fig 1 (see S2 Table for annual rates with 95% confidence intervals and S1 Fig for annual rates for specific NTDS). Hospitalization rates varied markedly over time, largely driven by fluctuations in dengue/chik. Table 2 shows joinpoint trends in age-standardized hospitalization rates per 100,000 population for the 5 most frequent endemic NTDs in Ecuador between 2000 and 2024. The average annual percentage change (AAPC) for all NTDs was 2.66% (95% CI 0.75-5.03) and for dengue/chik 7.76% (95% CI 4.23-12.32), with the other NTDs showing a downward trend.

**Table 2:** Joinpoint trends in age-standardized hospitalization rates (per 100,000 population) for all 21 neglected tropical diseases (NTDs) and the 5 most frequent endemic NTDs in Ecuador between 2000 and 2024.

NTD	Period, 2000–2024		Trend 1			Trend 2			Trend 3		
	AAPC (95% CI)	P value	Years	APC (95% CI)	P value	Years	APC (95% CI)	P value	Years	APC (95% CI)	P value
All 21 NTDs	2.66 (0.74-5.03)	0.018	2000-2015	2.72 (0.22-7.86)	0.037	2015-2018	-29.60 (-39.13—7.22)	0.007	2018-2024	23.78 (11.61-75.51)	0.008
Dengue and chikungunya	7.76 (4.23 – 12.32)	0.002	2000-2015	8.16 (3.22 – 18.97)	0.008	2015-2018	-43.75 (-56.48 –8.13)	0.009	2018-2024	47.78 (22.36 – 160.11)	0.006
Snakebite envenoming	-3.45 (-4.34 - -2.62)	<0.001	2000-2014	-0.21 (-1.64 – 1.93)	0.835	2014-2024	-7.81 (-11.27 - -5.69)	<0.001			
Soil-transmitted helminths	-5.62 (-6.56 –4.68)	<0.001	2000-2024	-5.62 (-6.56 –4.68)	<0.001						
Taeniasis and cysticercosis	-8.89 (-10.47 - -7.57)	<0.001	2000-2003	2.62 (-9.21 – 27.96)	0.732	2003-2024	-10.42 (-14.50 - -9.68)	0.002			
Scabies and ectoparasitoses	1.69 (0.62 – 3.18)	0.023	2000-2003	13.63 (-3.33 – 39.11)	0.072	2003-2007	-14.53 (-23.28 – 4.13)	0.065	2007-2024	3.88 (2.29 – 5.73)	0.022

The Annual Percent Change (APC) and Average Annual Percentage Change (AAPC) is significantly different from zero (P < 0.05).

CI – confidence interval. Age-standardized rates are expressed per 100,000 population using the WHO standard population.

**Fig 2:**
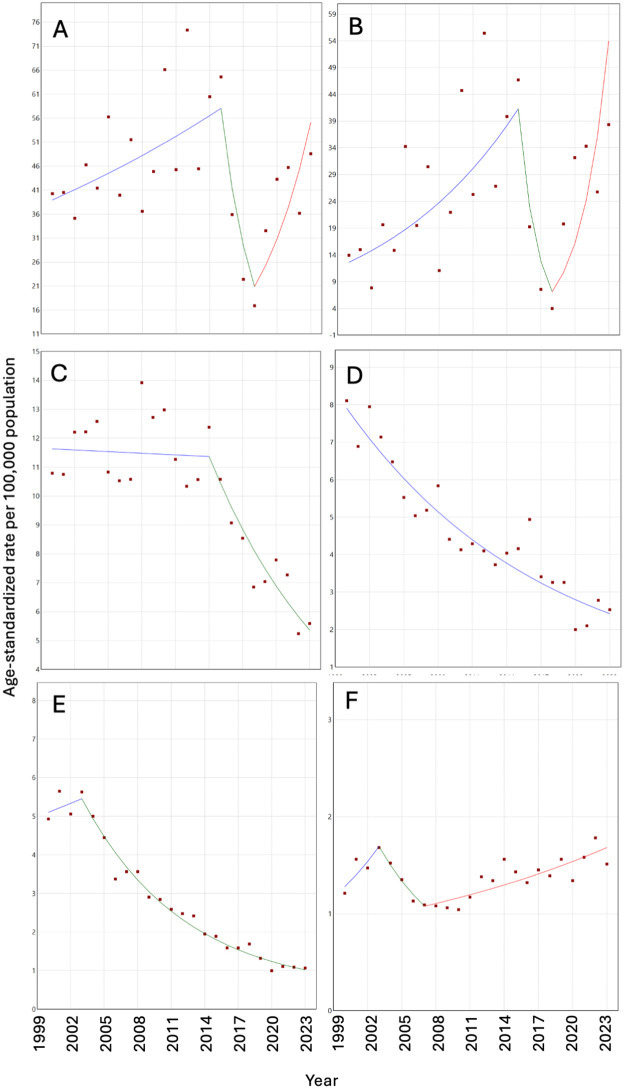
Joinpoint regression analysis of age-standardized hospitalization rates for neglected tropical diseases (NTDs) in Ecuador, 2000–2024. Panels show annual age-standardized hospitalization rates (per 100,000 population) and fitted joinpoint regression models for (A) all NTDs and (B–F) the five most frequent endemic NTDs: dengue/chikungunya, snakebite envenoming, soil-transmitted helminthiases, taeniasis/cysticercosis, and scabies/ectoparasitoses. Points represent observed rates and lines represent fitted log-linear regression segments. Joinpoints indicate statistically significant changes in trend. Segment-specific annual percentage changes (APCs) describe the direction and magnitude of trends within each period, and statistical significance was defined as P < 0.05. Rates were calculated using the WHO standard population.

Trends in hospitalization rates for all NTDs and for the five most common endemic NTDs with joinpoint regression lines are shown in Fig 2. Trends in hospitalization rates were strongly affected by arboviral hospitalization rates (Fig 2A and 2B) with an increase observed over the period 2000–2015 (dengue/chik, APC 8.16, 95% CI 3.22-18.97), followed by a decline between 2015 and 2018 (dengue/chik, APC -43.75, 95% CI -56.48 - -8.13), and a large increase between 2018 and 2024 (dengue/chik, APC 47.78, 95% CI 22.36-160.11). Snakebite envenoming showed a significant decrease between 2014 and 2024 (APC -7.81, 95% CI -11.27- -5.69) (Fig 2C), soil-transmitted helminths declined over the whole observation period (AAPC -5,62, 95% CI -6.56 - -4.68) (Fig 2D), while taeniasis and cysticercosis declined significantly between 2003 and 2024 (APC, -10.42, 95% CI -14.5 - -9.68) (Fig 2E). Scabies and other ectoparasitoses appeared to show a significant increase from 2007 (APC, 3.88, 95% CI 2.29-5.73) (Fig 2F). Annual reported number of hospitalizations attributed to non-endemic NTDs are shown in S3 Table with area of usual residence for patients with these NTDs provided in S4 Table.

**Fig 3:**
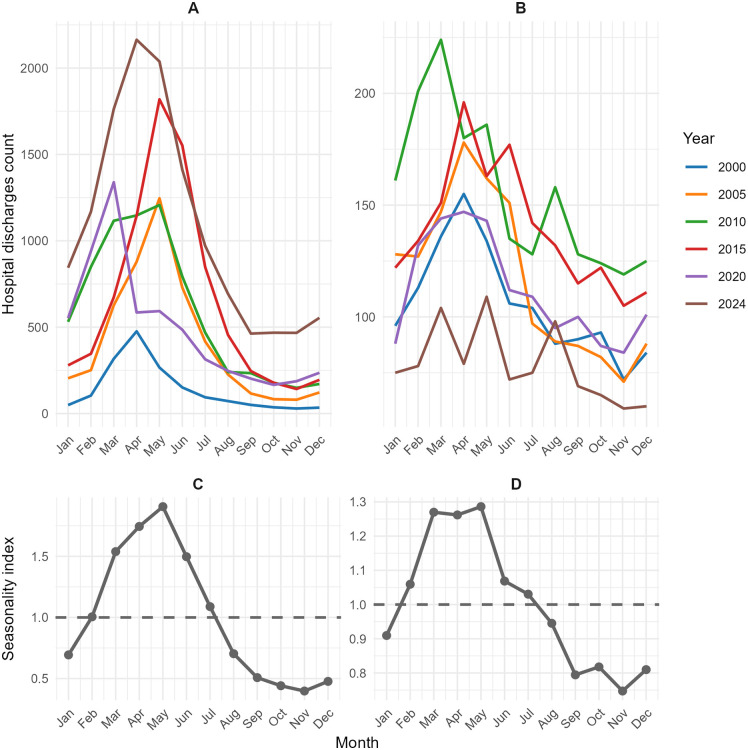
Monthly variation and seasonality of hospitalizations for dengue/chikungunya and snakebite in Ecuador, 2000–2024. Panels A and B show monthly hospitalization counts for dengue/chikungunya and snakebite, respectively, presented at five-year intervals. Panels C and D show the corresponding seasonality indices. The seasonality index was calculated separately for each five-year interval as the ratio of the mean number of hospitalizations in each calendar month to the overall mean monthly count within that interval. Values >1 indicate above-average activity, values <1 indicate below-average activity, and a value of 1 indicates that the monthly frequency equals the annual average. These plots illustrate consistent seasonal patterns, with higher hospitalization activity during wetter months.

Fig 3 shows how hospitalization frequencies for dengue/chik (Fig 3A) and snakebite (Fig 3B) varied monthly, reported at five-year intervals between 2000 and 2024, as well as the seasonality indices for these hospitalizations (Fig 3C and 3D, respectively). Clear seasonality (index >1) was observed for dengue/chik between March and July and for snakebite between February and July. The other endemic NTDs showed no clear evidence of seasonality.

**Fig 4:**
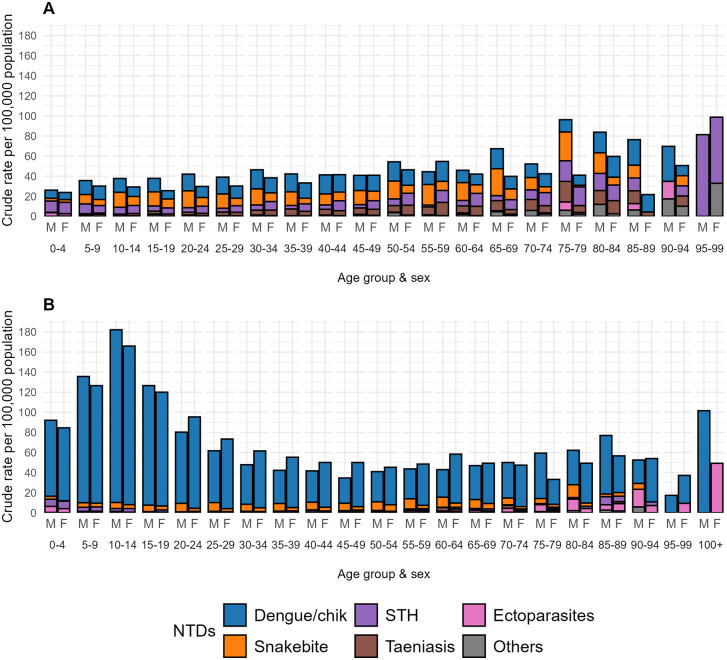
Age- and sex-specific crude hospitalization rates for endemic neglected tropical diseases (NTDs) in Ecuador in 2000 and 2024. Crude hospitalization rates (per 100,000 population) are shown by age group and sex for (A) 2000 and (B) 2024. M = male, F = female. Data are presented for the five most frequent endemic NTDs (Dengue and chikungunya; Snakebite envenoming; Soil-transmitted helminths; Taeniasis and cysticercosis; Scabies and other ectoparasitoses) and a grouped category of other endemic NTDs (including Chagas disease, echinococcosis, leishmaniasis, leprosy, onchocerciasis, yaws, foodborne trematodiases, mycetoma, chromoblastomycosis and other deep mycoses, and rabies). These distributions illustrate changes over time in the demographic profile of severe NTD-related morbidity requiring hospitalization. Hospitalization rates stratified by sex and age group for each province were calculated using population projections in 2000 and 2024 from INEC [36].

**Fig 5:**
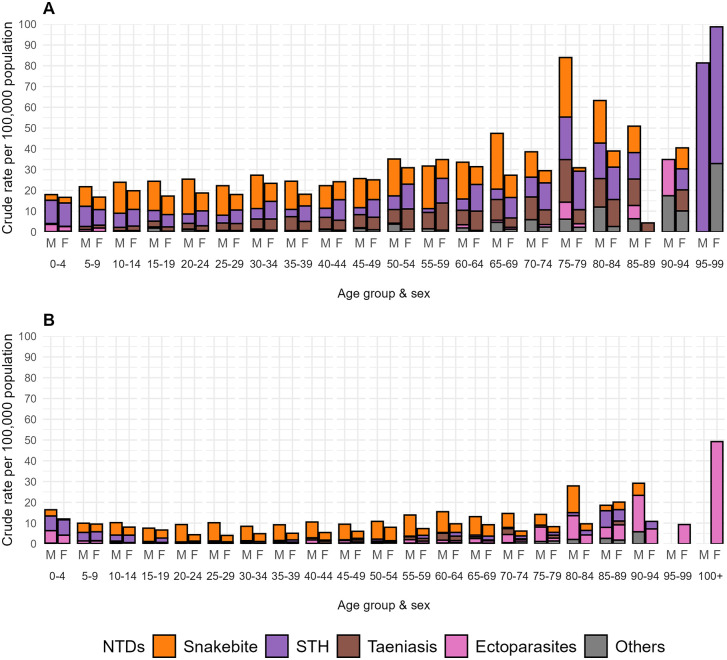
Age- and sex-specific crude hospitalization rates for non-arboviral endemic neglected tropical diseases (NTDs) in Ecuador in 2000 and 2024. Crude hospitalization rates (per 100,000 population) are shown by age group and sex for (A) 2000 and (B) 2024. Data are presented for the most frequent non-arboviral NTDs, including snakebite envenoming, soil-transmitted helminthiases, taeniasis/cysticercosis, scabies/ectoparasitoses, and a grouped category of other endemic NTDs (including Chagas disease, echinococcosis, leishmaniasis, leprosy, onchocerciasis, yaws, foodborne trematodiases, mycetoma, chromoblastomycosis and other deep mycoses, and rabies). The figure highlights age- and sex-specific differences in hospitalization patterns for non-arboviral NTDs over time.

Fig 4A and 4B shows crude hospitalization rates for 14 endemic NTDs by sex and age group in 2000 and 2024, respectively, while Fig 5A and 5B show these distributions for non-arboviral NTDs. Greater rates of endemic NTDs were observed in 2024, largely among those aged below 30 years, but these greater rates were attributed to the arboviral infections. Non-arboviral NTD hospitalization rates tended to increase with age and were reduced across all ages in 2024 compared to 2000, and rates appeared to be greater in men. Scabies and other ectoparasitoses had emerged by 2024 as an important cause of NTD hospitalizations, particularly among the elderly.

Data for the hospitalizations caused by endemic NTDs by Province in 2000 and 2024 are shown in S2 Fig. In 2000, rates were highest in the Amazon provinces (Morona Santiago Napo, Orellana, Pastaza, and Zamora Chinchipe) with particularly high rates of snakebite. A similar pattern was observed in 2024.

**Fig 6:**
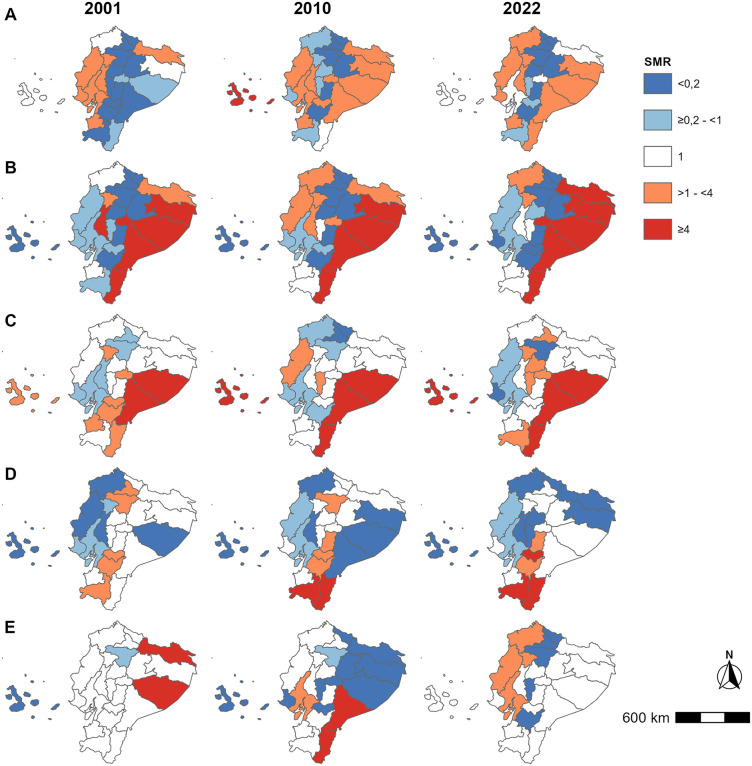
Spatial distribution of standardised morbidity ratios (SMRs) for five most frequent NTDs in Ecuador by province for censuds years of 2001, 2010, and 2022. SMRs were calculated using indirect standardization as the ratio of observed to expected hospitalizations, adjusted for age and sex. Values >1 indicate higher-than-expected morbidity and values <1 indicate lower-than-expected morbidity. Maps for the three census years, show (A) dengue/chikungunya, (B) snakebite envenoming, (C) soil-transmitted helminthiases, (D) taeniasis/cysticercosis, and (E) scabies/ectoparasitoses. Base map: Natural Earth (public domain) [38]. Maps were generated in R version 4.5.0 using the *sf*, *rnaturalearth*, *ggplot2*, and *ggspatial* packages. For visualization purposes, the Galápagos Islands were repositioned relative to mainland Ecuador to improve figure clarity.

We estimated standardized morbidity ratios for the 5 most frequent endemic NTDs for each of the 3 census years by province (2001, 2010, 2022). The data are provided in S5 Table and illustrated graphically in Fig 6. Relative morbidity was shown as white (expected level), shades of increasing blue (increasingly lower than expected), and shades of increasing red (increasingly greater than expected). Dengue/chik (Fig 6A) hospitalizations were lower than expected in the Andean provinces over the 3 census years and were consistently greater than expected in Coastal provinces and over time in the Amazon provinces. For snakebite (Fig 6B) and STH (Fig 6C), the Amazon region had a much higher than expected risk while the Andean provinces had a lower risk over the observation period. In the case of taeniasis/cysticercosis (Fig 6D), the risk was consistently higher in Andean, particularly southern Andean provinces. Ectoparasitoses (Fig 6E) showed less clear trends with excess morbidity risk shifting from central and northern Amazon provinces in 2001 to southern Amazon/Andean provinces in 2010, to central and northern coastal provinces in 2022.

## Discussion

This analysis of national hospitalization data provides the most comprehensive assessment to date of long-term trends in NTD-related hospitalizations in Ecuador, spanning 25 years from 2000 to 2024. NTDs accounted for 0.7% of all hospitalizations, representing a relatively small proportion of the overall burden but likely reflecting the more severe end of the clinical spectrum requiring inpatient care. By comparison, ambulatory care–sensitive conditions in children under 5 years accounted for 26.6% of hospital discharges in Ecuador during 2000–2023 [39]. Overall, NTD hospitalizations increased over time, driven primarily by arboviral morbidity (dengue and chikungunya), while hospitalizations attributable to non-arboviral NTD declined.

The vast majority (97.1%) of NTD hospitalizations were attributable to five NTDs—arboviral infections (dengue/chik), snakebite envenoming, soil-transmitted helminthiases, taeniasis/cysticercosis, and ectoparasitoses — and is consistent with regional patterns observed elsewhere in Latin America, including in northeastern Brazil, although with notable differences in disease burden composition. For example, while Brazil’s NTD hospitalization burden saw a decline of 10.3% over the period, 2001–2018, it prominently included leishmaniasis (8.6%) and leprosy (6.4%) [40], diseases that accounted for <1% of Ecuador’s NTD-related hospitalizations. Hospitalization rates for leprosy in Ecuador have declined from a rate of 1.33 (95% CI 1.06-1.60) per 100,000 in 2000 to 0.06 (0.01-0.11) in 2024 reflecting a declining disease incidence [41], while the cutaneous form of leishmaniasis that is endemic in Ecuador rarely requires hospitalization [42].

Arboviral infections (dengue/chik) represented the largest and fastest-growing share of hospitalizations, with an AAPC that increased steeply after 2018 (+47.8%). This rise reflects regional dengue trends [43]. The decline in dengue/chik hospitalizations around 2017 is consistent with patterns observed elsewhere in Latin America that has been explained by several factors including increased population immunity from previous years of high transmission, potential cross-protection from the Zika virus, and enhanced vector control efforts [44,45]. Sharp changes in dengue/chikungunya trends should be interpreted with caution when confidence intervals are wide, particularly in recent periods (2015–2018 and 2018–2024), as these may reflect unstable AAPC estimates due to small case numbers, short time intervals, or substantial year-to-year variability. Seasonality analysis confirmed well-documented transmission patterns, with peaks in the rainy season, in agreement with previous studies from Ecuador and other regions of Latin America [21,46].

While arbovirus-related morbidity requiring hospitalization is increasing, that attributed to other non-arboviral NTDs such as STH, taeniasis/cysticercosis, and snakebite showed significant and sustained declines. For example, STH-related hospitalization decreased (AAPC -5.62%), suggesting an effect of deworming and improved sanitation, although reductions were not uniform across demographic groups. Severe STH morbidity resulting in hospitalization results from complications of heavy parasite burdens such as intestinal obstruction in children caused by *Ascaris lumbricoides*, a leading cause of surgical emergencies [47], or severe anaemia consequent to hookworm infections [48]. However, trends may also be influenced by changes in admission practices, access to care, outpatient management, or coding. A plausible explanation is that deworming programmes and improving access to clean water and sanitation have led to reduced worm burdens, thereby lowering the proportions of infected individual at risk of severe morbidity [19,49]. Women had higher hospitalization rates for STH than men, particularly from age 10 onward, consistent with prior studies indicating maternal and early childhood STH transmission risks [50,51].

Ectoparasitoses, particularly scabies, emerged as a growing cause of hospitalization, especially among the elderly. This aligns with trends seen in high-income countries such as Spain and South Korea [52,53]. The increase highlights the importance of addressing geriatric vulnerability to NTDs and associated socio-environmental risk factors such as overcrowding, poverty, and poor hygiene. In addition, increasing violence in several Coastal provinces may further limit access to healthcare services and contribute to delayed diagnosis and treatment.

Sex and age disparities were notable across NTDs. Snakebite disproportionately affected young males, as reported elsewhere across LMICs [54], likely due to occupational exposure in agriculture and forest-related work. Conversely, the rise in hospitalizations among older adults—especially from STH and ectoparasitoses—emphasizes the need for age-sensitive interventions and surveillance.

Spatial analyses using standardized morbidity ratios (SMRs) revealed substantial geographic heterogeneity in NTD-related hospitalizations across Ecuador. The Amazon region consistently exhibited higher relative morbidity for several NTDs, particularly snakebite, soil-transmitted helminthiases, and arboviral infections, and is characterized by higher levels of poverty, rurality, and a greater proportion of Indigenous populations [28], all of which may increase vulnerability to severe disease. Over time, patterns evolved, with a relative shift in arboviral SMRs from Coastal regions in earlier years toward the Amazon by 2022. Persistently elevated SMRs for taeniasis/cysticercosis in southern Andean provinces are consistent with long-standing epidemiological patterns linked to traditional pork consumption and limited meat inspection [25].

Multiple factors may explain these patterns. A true shift in underlying risk is plausible and may reflect population mobility, urban expansion, environmental change, and broader social determinants, including poverty, housing conditions, and demographic changes such as population ageing, which may increase vulnerability among older adults. However, regional and temporal differences in healthcare access, diagnostic capacity, and reporting completeness are also likely to have influenced observed patterns. Limited access to laboratory confirmation and specialist care in remote regions may contribute to under-recognition, while improvements in diagnostic capacity and clinician awareness may have increased case detection in better-resourced settings. Conversely, earlier patterns observed in Amazon provinces may reflect underreporting or structural inequalities in access to care.

In addition, COVID-19–related disruptions—including changes in healthcare-seeking behaviour, temporary reallocation of hospital resources, and interruptions to routine services—may have influenced NTD-related hospitalization patterns and reporting in recent years. Together, these findings suggest that observed spatial and temporal variation reflects a combination of epidemiological change and health system factors rather than differences in transmission alone.

Despite reported progress toward elimination of certain NTDs in Ecuador, including yaws [18] and onchocerciasis (certified in 2015) [16], hospitalizations were still recorded for these and other “non-endemic” NTDs, including Buruli ulcer and noma. Such observations most likely reflect diagnostic misclassification or coding inaccuracies, although imported infections cannot be excluded. Diagnostic overlap with clinically similar conditions may also contribute – for example cutaneous leishmaniasis and treponematoses may cause diagnostic confusion, while chronic intestinal schistosomiasis may present with non-specific gastrointestinal symptoms. Importantly, hospital discharge data do not capture whether diagnoses were subsequently confirmed through laboratory or specialist evaluation. In the case of schistosomiasis and lymphatic filariasis (S3 Table), although published reports using these official data sources are available [55,56], to our knowledge, no case of autochthonous transmission of either infection (i.e., *Schistosoma* spp. and *Wuchereria bancrofti*) has been documented (i.e., with appropriate identification of the causative pathogen) in the country. The low number of recorded cases of most of these non-endemic NTDs—typically fewer than two per year for some conditions—supports the interpretation that these do not represent sustained local presence. These findings highlight limitations in national health information systems and underscore the need for improved diagnostic protocols, ICD-10 coding practices, and data quality assurance to strengthen surveillance accuracy. Some of these cases, if real, may represent imported cases from other regions of Latin America or where these diseases are known to occur. Ecuador is crossed by the land routes of a growing influx of migrants on their way to the North. However, almost all these cases were documented among individuals with a normal residence in Ecuador (S4 table) and there was no evidence of an increase in cases over time.

Although the overall in-hospital case fatality rate for NTD hospitalizations was low (0.4%), important disease-specific disparities were observed. Chagas (6.7%) and leprosy (3.8%) accounted for the highest case-fatality rates, in line with findings from Brazil and other countries [57]. The anomalous mortality rate given for rabies (8.4%) likely represents the reason for hospital admission (e.g., patients with animal bites considered at risk of rabies) rather than diagnosis at discharge. An isolated outbreak of rabies (causing non-hospitalized deaths) transmitted through the bites of hematophagous bats was reported from Indigenous communities in the Amazon region in 2011 [58]. Ecuador’s in-hospital dengue case fatality rate was 0.3%. This estimate is not directly comparable to national (<0.04%) or WHO (<0.5% by 2025) targets, which refer to overall case fatality rather than in-hospital mortality. Nevertheless, in-hospital fatality remains relatively high, suggesting potential gaps in timely care or clinical management, consistent with evidence from Latin America indicating that mortality among hospitalized dengue patients is influenced by comorbidities, disease severity at presentation, and health system factors [59,60].

This study has several limitations. It relies on hospitalization data alone, without access to outpatient or surveillance datasets that are not yet publicly available, and lacks diagnostic validation or comorbidity information within the national registry database. Hospitalization data should be interpreted in the light of potential misclassification due to ICD-10 coding and the inherent limitations of the ecological design. The use of hospital discharge data captures only cases requiring hospitalisation, reflecting health-seeking behaviour, admission practices, diagnostic capacity, and health system constraints rather than true population incidence. As a result, the observed trends may be influenced by changes in access to care and reporting practices over time and should be interpreted as representing a combination of epidemiological patterns and health system dynamics, rather than incidence alone. Further, there appear to be important issues with data quality control with no independent verification of ICD-10 code accuracy or diagnostic validity. Although ICD-10 coding for dengue evolved over time, we mitigated potential bias by aggregating dengue-related diagnoses and found no evidence of structural breaks; however, residual misclassification cannot be excluded. Early coding may have been influenced by transitional use of legacy classifications and evolving familiarity with the WHO 2009 framework. Temporal trends may also have been influenced by changes in health system organization (e.g., the 2012 health reform), access to care, and clinical management over time. In addition, improvements in diagnostic capacity, clinician awareness, and coding completeness over time may have contributed to temporal changes independent of true disease trends. Some NTDs require confirmation through laboratory or imaging testing - such capacity is restricted to the larger cities and is not readily available in district hospitals in many parts of the country. Additionally, demographic variables such as ethnicity and rurality have only been available since 2014–2015, restricting our ability to consider these factors. Available data were aggregated and the analysis ecological, thus limiting our ability to account for multiple hospitalizations of the same individuals or infer from group-level associations to those of individuals. Although SMRs adjust for demographic differences, they remain limited by residual confounding, reliance on the reference population, and reduced comparability when underlying risks differ across populations. Despite these limitations, the study offers important insights into spatiotemporal patterns of severe NTD-related morbidity in Ecuador that are relevant to informing public health policies and resource allocation. However, comprehensive assessment of progress towards SDG target 3.3 will require integration of hospitalization data with national surveillance systems to capture the full spectrum of disease.

In conclusion, our data show increasing hospitalizations attributed to arboviral infections and ectoparasites, alongside declining rates attributable to helminthiases and zoonotic parasitic infections. The latter may suggest gradual improvements in health and sanitation infrastructure in the country since 2000. Hospitalization trends can inform health system planning, such as resource allocation for severe dengue management or snakebite care in high-risk provinces. Comparisons in hospitalization rates between provinces and by age showed a relatively greater burden of more severe morbidity and healthcare utilization attributable to NTDs in the Amazon region and among vulnerable age groups. These patterns should be interpreted with caution, as disparities in healthcare access and reporting may partly explain spatial differences, particularly elevated SMRs in remote Amazonian provinces, consistent with the uneven expansion of hospital beds favouring the Coastal and Andean regions over the Amazon. Documenting progress towards SDG 3.3 will require the national surveillance and primary care databases to be made available to the academic community. Interventions to control and eliminate NTDs likely will need to be geographically and demographically tailored—particularly to Amazonian populations and vulnerable age groups—and supported by stronger surveillance and more robust notification processes with improved diagnostic capacity at primary care level, improved data collection and registration practices, and sustained health system investment.

## Supporting information

S1 Table ICD-10 codes used for the 21 neglected tropical diseases. (DOCX)

S2 Table Age-standardized hospitalization rates for neglected tropical diseases (NTDs) in Ecuador between 2000 and 2024. Shown are rates per 100,000 population and 95% confidence intervals.
(DOCX)

S3 Table Frequency of hospital discharges for 7 non-endemic NTDs with corresponding hospitalizations reported in Ecuador between 2000 and 2024. (DOCX)

S4 Table Frequencies and origins (area of usual residence) of patients hospitalized with non-endemic NTDs in Ecuador between 2015 and 2024 for which data are available. (DOCX)

S5 Table Standardized morbidity ratio (and 95% confidence intervals) of hospitalization rates attributed to 5 most frequent endemic neglected tropical diseases in Ecuador by geoclimatic region and province in census years of 2001, 2010, and 2022. (DOCX)

S1 Fig Age-standardized hospitalization rates for 14 endemics neglected tropical diseases (NTDs) in Ecuador between 2000 and 2024 (data are from S2_Table). (TIFF)

S2 Fig Crude cumulative hospitalization rates (per 100,000 population) for endemic NTDs by province in Ecuador between 2000 and 2024. (TIFF)

S1 Data Raw data used for analyses. (XLSX)
