# Supplementary material for: Spatiotemporal trends of neglected tropical disease hospitalizations in Ecuador over 25-years from 2000 to 2024
Source: PLoS Negl Trop Dis. 2026 May 18;20(5):e0013688. doi: 10.1371/journal.pntd.0013688 (PMC13197067; doi:10.1371/journal.pntd.0013688)
Supplement: S1 Table — (DOCX) [file pntd.0013688.s001.docx]

S1 Table. ICD-10 codes for the 21 neglected tropical diseases

| Disease | ICD 10 Code | Naming |
| --- | --- | --- |
| Dengue and chikungunya |  |  |
|  | A90 | Dengue fever [classical dengue] |
|  | A91 | Dengue haemorrhagic fever |
|  | A920 | Chikungunya virus disease |
|  | A928 | Other specified mosquito-borne viral fevers |
|  | A929 | Mosquito-borne viral fever. unspecified |
|  | A97* | Dengue |
|  | A970* | Dengue without warning signs |
|  | A971* | Dengue with warning signs |
|  | A972* | Severe Dengue |
|  | A979 | Dengue. unspecified |
|  | A99 | Unspecified viral haemorrhagic fever |
| Dracunculiasis |  |  |
|  | B72 | Dracunculiasis |
| Chagas disease | | |
|  | B57 | Chagas disease |
|  | B570 | Acute Chagas disease with heart involvement |
|  | B571 | Acute Chagas disease without heart involvement |
|  | B572 | Chagas disease (chronic) with heart involvement |
|  | B573 | Chagas disease (chronic) with digestive system involvement |
|  | B574 | Chagas disease (chronic) with nervous system involvement |
|  | B575 | Chagas disease (chronic) with other organ involvement |
|  | I412 | Myocarditis in other infectious and parasitic diseases classified elsewhere: Chagas disease (chronic) |
|  | I981 | Cardiovascular disorders in other infectious and parasitic diseases classified elsewhere: involvement NEC. in Chagas disease (chronic) |
| Snakebite envenoming |  |  |
|  | T63 | Toxic effect of contact with venomous animals |
|  | T630 | Snake venom |
|  | T638 | Toxic effect of contact with other venomous animals |
|  | T639 | Toxic effect of contact with unspecified venomous animal |
| Echinococcosis |  |  |
|  | B67 | Echinococcosis |
|  | B670 | Echinococcus granulosus infection of liver |
|  | B671 | Echinococcus granulosus infection of lung |
|  | B672 | Echinococcus granulosus infection of bone |
|  | B673 | Echinococcus granulosus infection. other and multiple sites |
|  | B674 | Echinococcus granulosus infection. unspecified |
|  | B675 | Echinococcus multilocularis infection of liver |
|  | B676 | Echinococcus multilocularis infection. other and multiple sites |
|  | B677 | Echinococcus multilocularis infection. unspecified |
|  | B678 | Echinococcosis. unspecified. of liver |
|  | B679 | Echinococcosis. other and unspecified |
| Schistosomiasis |  |  |
|  | B65 | Schistosomiasis [bilharziasis] |
|  | B650 | Schistosomiasis due to Schistosoma haematobium [urinary schistosomiasis] |
|  | B651 | Schistosomiasis due to Schistosoma mansoni [intestinal schistosomiasis] |
|  | B652 | Schistosomiasis due to Schistosoma japonicum |
|  | B653 | Cercarial dermatitis |
|  | B658 | Other schistosomiases |
|  | B659 | Schistosomiasis. unspecified |
| Lymphatic filariasis |  |  |
|  | B74 | Filariasis |
|  | B740 | Filariasis due to Wuchereria bancrofti |
|  | B741 | Filariasis due to Brugia malayi |
|  | B742 | Filariasis due to Brugia timori |
|  | B743 | Loiasis |
|  | B744 | Mansonelliasis |
|  | B748 | Other filariases |
|  | B749 | Filariasis. unspecified |
| Soil-transmitted helminthiases |  |  |
|  | B78 | Strongyloidiasis |
|  | B780 | Intestinal strongyloidiasis |
|  | B781 | Cutaneous strongyloidiasis |
|  | B787 | Disseminated strongyloidiasis |
|  | B789 | Strongyloidiasis. unspecified |
|  | B76 | Hookworm diseases |
|  | B760 | Ancylostomiasis |
|  | B761 | Necatoriasis |
|  | B768 | Other hookworm diseases |
|  | B769 | Hookworm disease. unspecified |
|  | B77 | Ascariasis |
|  | B770 | Ascariasis with intestinal complications |
|  | B778 | Ascariasis with other complications |
|  | B779 | Ascariasis. unspecified |
|  | B79 | Trichuriasis |
|  | B82 | Unspecified intestinal parasitism |
|  | B820 | Intestinal helminthiasis. unspecified |
|  | B829 | Intestinal parasitism. unspecified |
| Leishmaniasis |  |  |
|  | B55 | Leishmaniasis |
|  | B550 | Visceral leishmaniasis |
|  | B551 | Cutaneous leishmaniasis |
|  | B552 | Mucocutaneous leishmaniasis |
|  | B559 | Leishmaniasis. unspecified |
| Leprosy |  |  |
|  | A30 | Leprosy [Hansen disease] |
|  | A300 | Indeterminate leprosy |
|  | A301 | Tuberculoid leprosy |
|  | A302 | Borderline tuberculoid leprosy |
|  | A303 | Borderline leprosy |
|  | A304 | Borderline lepromatous leprosy |
|  | A305 | Lepromatous leprosy |
|  | A308 | Other forms of leprosy |
|  | A309 | Leprosy. unspecified |
|  | B92 | Sequelae of leprosy |
| Mycetoma. chromoblastomycosis and other deep mycoses |  |  |
|  | B43 | Chromomycosis and phaeomycotic abscess |
|  | B430 | Cutaneous chromomycosis |
|  | B431 | Phaeomycotic brain abscess |
|  | B432 | Subcutaneous phaeomycotic abscess and cyst |
|  | B438 | Other forms of chromomycosis |
|  | B439 | Chromomycosis. unspecified |
|  | B47 | Mycetoma |
|  | B470 | Eumycetoma |
|  | B471 | Actinomycetoma |
|  | B479 | Mycetoma. unspecified |
| Onchocerciasis |  |  |
|  | B73 | Onchocerciasis |
| Yaws |  |  |
|  | A66 | Yaws |
|  | A660 | Initial lesions of yaws |
|  | A661 | Multiple papillomata and wet crab yaws |
|  | A662 | Other early skin lesions of yaws |
|  | A663 | Hyperkeratosis of yaws |
|  | A664 | Gummata and ulcers of yaws |
|  | A665 | Gangosa |
|  | A666 | Bone and joint lesions of yaws |
|  | A667 | Other manifestations of yaws |
|  | A668 | Latent yaws |
|  | A669 | Yaws. unspecified |
| Rabies |  |  |
|  | A82 | Rabies |
|  | A820 | Sylvatic rabies |
|  | A821 | Urban rabies |
|  | A829 | Rabies. unspecified |
| Scabies and other ectoparasitoses |  |  |
|  | B86 | Scabies |
|  | B85 | Pediculosis and phthiriasis |
|  | B850 | Pediculosis due to Pediculus humanus capitis |
|  | B851 | Pediculosis due to Pediculus humanus corporis |
|  | B852 | Pediculosis. unspecified |
|  | B853 | Phthiriasis |
|  | B854 | Mixed pediculosis and phthiriasis |
|  | B87 | Myiasis |
|  | B870 | Cutaneous myiasis |
|  | B871 | Wound myiasis |
|  | B872 | Ocular myiasis |
|  | B873 | Nasopharyngeal myiasis |
|  | B874 | Aural myiasis |
|  | B878 | Myiasis of other sites |
|  | B879 | Myiasis. unspecified |
|  | B881 | Tungiasis [sandflea infestation] |
| Taeniasis and cysticercosis |  |  |
|  | B68 | Taeniasis |
|  | B680 | Taenia solium taeniasis |
|  | B681 | Taenia saginata taeniasis |
|  | B689 | Taeniasis. unspecified |
|  | B69 | Cysticercosis |
|  | B690 | Cysticercosis of central nervous system |
|  | B691 | Cysticercosis of eye |
|  | B698 | Cysticercosis of other sites |
|  | B699 | Cysticercosis. unspecified |
| Trachoma |  |  |
|  | A71 | Trachoma |
|  | A710 | Initial stage of trachoma |
|  | A711 | Active stage of trachoma |
|  | A719 | Trachoma. unspecified |
|  | B940 | Sequelae of trachoma |
| Foodborne trematodiases |  |  |
|  | B66 | Other fluke infections |
|  | B660 | Opisthorchiasis |
|  | B661 | Clonorchiasis |
|  | B663 | Fascioliasis |
|  | B664 | Paragonimiasis |
|  | B668 | Other specified fluke infections |
|  | B669 | Fluke infection. unspecified |
| Human African trypanosomiasis |  |  |
|  | B56 | African trypanosomiasis |
|  | B560 | Gambiense trypanosomiasis |
|  | B561 | Rhodesiense trypanosomiasis |
|  | B569 | African trypanosomiasis. unspecified |
| Buruli ulcer |  |  |
|  | A311 | Cutaneous mycobacterial infection Buruli ulcer |
| Noma |  |  |
|  | A690 | Necrotizing ulcerative stomatitis Noma |

*Implementation date January 2016
