# Supplementary material for: Spatiotemporal trends of neglected tropical disease hospitalizations in Ecuador over 25-years from 2000 to 2024
Source: PLoS Negl Trop Dis. 2026 May 18;20(5):e0013688. doi: 10.1371/journal.pntd.0013688 (PMC13197067; doi:10.1371/journal.pntd.0013688)
Supplement: S2 Table — Shown are rates per 100,000 population and 95% confidence intervals. (DOCX) [file pntd.0013688.s002.docx]

S2 Table. Age-standardized hospitalization rates for NTDs in Ecuador between 2000 and 2024. Shown are rates per 100,000 population and 95% confidence intervals.

| NTDs | Age-standardized rate per 100.000 (95% CI) | | | | | | | | | | | | | | | | | | | | | | | | |
| --- | --- | --- | --- | --- | --- | --- | --- | --- | --- | --- | --- | --- | --- | --- | --- | --- | --- | --- | --- | --- | --- | --- | --- | --- | --- |
|  | 2000 | 2001 | 2002 | 2003 | 2004 | 2005 | 2006 | 2007 | 2008 | 2009 | 2010 | 2011 | 2012 | 2013 | 2014 | 2015 | 2016 | 2017 | 2018 | 2019 | 2020 | 2021 | 2022 | 2023 | 2024 |
| All NTDs | 41.22 (37.45;45) | 41.5 (37.82;45.19) | 35.87 (32.49;39.25) | 47.19 (43.58;50.8) | 42.46 (38.87;46.06) | 57.22 (53.59;60.85) | 40.7 (37.59;43.81) | 52.44 (49.03;55.85) | 37.41 (34.25;40.57) | 45.58 (42.41;48.76) | 67.42 (63.79;71.05) | 46.14 (42.92;49.35) | 75.2 (71.75;78.66) | 46.17 (43.16;49.18) | 61.46 (58.07;64.84) | 65.34 (62.26;68.42) | 36.53 (33.95;39.1) | 22.98 (20.77;25.19) | 17.69 (15.56;19.83) | 33.27 (30.88;35.66) | 43.88 (41.64;46.12) | 46.37 (43.88;48.86) | 36.92 (34.51;39.33) | 49.04 (46.64;51.44) | 81.95 (79.19;84.71) |
| Endemic NTDs |  | | | | | | | | | | | | | | | | | | | | | | | | |
| Dengue and chikungunya | 14.03 (13.33;14.74) | 15.09 (14.39;15.8) | 7.95 (7.43;8.46) | 19.73 (18.95;20.5) | 14.95 (14.28;15.62) | 34.34 (33.35;35.32) | 19.59 (18.85;20.32) | 30.57 (29.66;31.47) | 11.19 (10.63;11.74) | 22.06 (21.3;22.82) | 44.84 (43.77;45.9) | 25.38 (24.59;26.17) | 55.53 (54.38;56.68) | 26.92 (26.12;27.72) | 39.97 (39.01;40.94) | 46.82 (45.78;47.86) | 19.36 (18.69;20.03) | 7.68 (7.25;8.1) | 4.1 (3.8;4.4) | 19.88 (19.22;20.53) | 32.26 (31.43;33.09) | 34.39 (33.54;35.25) | 25.86 (25.11;26.6) | 38.44 (37.53;39.34) | 71.79 (70.55;73.02) |
| Snakebite envenoming | 10.79 (10.16;11.41) | 10.75 (10.14;11.37) | 12.21 (11.56;12.86) | 12.22 (11.58;12.86) | 12.58 (11.94;13.22) | 10.83 (10.24;11.41) | 10.53 (9.97;11.1) | 10.58 (10.02;11.15) | 13.92 (13.27;14.57) | 12.72 (12.12;13.33) | 12.98 (12.38;13.58) | 11.27 (10.72;11.82) | 10.34 (9.82;10.87) | 10.57 (10.05;11.1) | 12.38 (11.82;12.94) | 10.58 (10.07;11.09) | 9.07 (8.6;9.54) | 8.54 (8.08;8.99) | 6.85 (6.45;7.25) | 7.04 (6.63;7.45) | 7.79 (7.37;8.21) | 7.27 (6.87;7.67) | 5.24 (4.9;5.58) | 5.59 (5.24;5.94) | 5.23 (4.89;5.56) |
| Soil-transmitted helminthiases | 7.65 (7.13;8.17) | 6.43 (5.96;6.9) | 7.49 (7;7.98) | 6.68 (6.22;7.14) | 6.02 (5.6;6.44) | 5.07 (4.69;5.45) | 4.58 (4.22;4.94) | 4.73 (4.37;5.09) | 5.38 (5.01;5.76) | 3.95 (3.63;4.26) | 3.67 (3.36;3.97) | 3.83 (3.52;4.13) | 3.64 (3.34;3.93) | 3.27 (2.99;3.54) | 3.58 (3.29;3.86) | 3.7 (3.41;4) | 4.48 (4.16;4.8) | 2.95 (2.69;3.21) | 2.8 (2.55;3.05) | 2.8 (2.55;3.05) | 1.54 (1.35;1.73) | 1.64 (1.44;1.83) | 2.32 (2.09;2.55) | 2.07 (1.86;2.29) | 1.88 (1.67;2.09) |
| Taeniasis and cysticercosis | 4.47 (4.05;4.9) | 5.19 (4.73;5.65) | 4.6 (4.18;5.03) | 5.17 (4.71;5.62) | 4.54 (4.12;4.95) | 3.99 (3.61;4.37) | 2.92 (2.6;3.24) | 3.11 (2.78;3.44) | 3.11 (2.79;3.44) | 2.45 (2.17;2.73) | 2.39 (2.12;2.66) | 2.13 (1.87;2.38) | 2.02 (1.77;2.26) | 1.96 (1.72;2.2) | 1.49 (1.28;1.69) | 1.43 (1.23;1.63) | 1.13 (0.96;1.3) | 1.13 (0.96;1.3) | 1.23 (1.04;1.41) | 0.86 (0.71;1.01) | 0.54 (0.42;0.66) | 0.65 (0.52;0.77) | 0.63 (0.49;0.76) | 0.61 (0.49;0.74) | 0.48 (0.37;0.59) |
| Scabies and other ectoparasitoses | 0.83 (0.63;1.02) | 1.18 (0.95;1.4) | 1.09 (0.9;1.28) | 1.3 (1.09;1.51) | 1.14 (0.93;1.36) | 0.97 (0.76;1.18) | 0.75 (0.57;0.93) | 0.71 (0.55;0.88) | 0.7 (0.53;0.87) | 0.68 (0.52;0.84) | 0.66 (0.49;0.82) | 0.79 (0.62;0.96) | 1 (0.82;1.17) | 0.96 (0.79;1.14) | 1.18 (0.99;1.37) | 1.05 (0.87;1.22) | 0.94 (0.78;1.1) | 1.07 (0.91;1.24) | 1.01 (0.84;1.17) | 1.18 (1.01;1.35) | 0.96 (0.79;1.13) | 1.2 (1.02;1.39) | 1.4 (1.22;1.59) | 1.13 (0.96;1.29) | 1.28 (1.11;1.46) |
| Leprosy | 1.33 (1.06;1.6) | 1.4 (1.09;1.71) | 1.01 (0.77;1.25) | 0.65 (0.45;0.85) | 1.02 (0.75;1.28) | 0.51 (0.32;0.69) | 0.89 (0.7;1.09) | 0.85 (0.65;1.05) | 0.99 (0.77;1.2) | 1.05 (0.82;1.28) | 0.93 (0.7;1.16) | 0.48 (0.31;0.65) | 0.35 (0.2;0.49) | 0.28 (0.16;0.39) | 0.33 (0.19;0.46) | 0.11 (0.03;0.18) | 0.06 (0;0.13) | 0.03 (-0.01;0.07) | 0.09 (0.02;0.16) | 0.01 (-0.01;0.03) | 0.08 (0.02;0.14) | 0.1 (0.03;0.16) | 0.07 (0.01;0.13) | 0.05 (0;0.09) | 0.06 (0.01;0.11) |
| Leishmaniasis | 0.83 (0.62;1.05) | 0.5 (0.37;0.63) | 0.73 (0.53;0.92) | 0.65 (0.45;0.86) | 0.91 (0.72;1.09) | 0.48 (0.32;0.64) | 0.66 (0.48;0.84) | 0.5 (0.33;0.67) | 0.4 (0.25;0.56) | 0.35 (0.21;0.48) | 0.62 (0.43;0.8) | 0.44 (0.31;0.57) | 0.47 (0.32;0.62) | 0.37 (0.23;0.5) | 0.38 (0.25;0.51) | 0.36 (0.24;0.49) | 0.32 (0.21;0.44) | 0.39 (0.27;0.52) | 0.39 (0.26;0.52) | 0.4 (0.28;0.52) | 0.16 (0.07;0.24) | 0.18 (0.1;0.26) | 0.31 (0.2;0.41) | 0.33 (0.21;0.44) | 0.29 (0.19;0.39) |
| Chagas disease | 0.42 (0.23;0.61) | 0.27 (0.13;0.42) | 0.22 (0.09;0.35) | 0.4 (0.23;0.57) | 0.57 (0.38;0.76) | 0.35 (0.18;0.52) | 0.19 (0.08;0.3) | 0.25 (0.11;0.38) | 0.3 (0.17;0.43) | 0.28 (0.16;0.4) | 0.29 (0.16;0.41) | 0.24 (0.12;0.36) | 0.28 (0.17;0.39) | 0.24 (0.15;0.34) | 0.31 (0.18;0.44) | 0.26 (0.14;0.37) | 0.3 (0.18;0.41) | 0.2 (0.1;0.29) | 0.17 (0.08;0.25) | 0.15 (0.07;0.23) | 0.12 (0.05;0.18) | 0.13 (0.05;0.2) | 0.13 (0.05;0.2) | 0.14 (0.07;0.2) | 0.1 (0.04;0.17) |
| Mycetoma. chromoblastomycosis and other deep mycoses | 0.17 (0.04;0.31) | 0.15 (0.04;0.25) | 0.03 (-0.01;0.07) | 0.03 (-0.01;0.07) | 0.12 (0.03;0.21) | 0.05 (0.01;0.1) | 0.07 (0.01;0.14) | 0.08 (0.02;0.14) | 0.13 (0.06;0.21) | 0.07 (0.01;0.13) | 0.25 (0.14;0.36) | 0.26 (0.15;0.36) | 0.27 (0.15;0.38) | 0.42 (0.28;0.55) | 0.54 (0.41;0.68) | 0.41 (0.29;0.53) | 0.43 (0.31;0.55) | 0.4 (0.28;0.52) | 0.3 (0.21;0.4) | 0.26 (0.16;0.37) | 0.2 (0.11;0.3) | 0.2 (0.12;0.28) | 0.31 (0.2;0.42) | 0.15 (0.09;0.22) | 0.27 (0.17;0.38) |
| Echinococcosis | 0.2 (0.06;0.34) | 0.05 (-0.02;0.12) | 0.1 (0.01;0.19) | 0.02 (-0.02;0.05) | 0.21 (0.12;0.31) | 0.11 (0.02;0.2) | 0.05 (-0.01;0.11) | 0.11 (0.02;0.19) | 0.1 (0.03;0.17) | 0.05 (-0.01;0.1) | 0.1 (0.02;0.18) | 0.03 (-0.01;0.07) | 0.07 (0.01;0.12) | 0.18 (0.09;0.27) | 0.17 (0.07;0.27) | 0.14 (0.05;0.22) | 0.09 (0.02;0.16) | 0.06 (0.01;0.11) | 0.18 (0.08;0.27) | 0.2 (0.11;0.29) | 0.09 (0.03;0.15) | 0.17 (0.09;0.26) | 0.16 (0.08;0.23) | 0.17 (0.09;0.26) | 0.21 (0.12;0.31) |
| Yaws | 0.01 (-0.01;0.04) | 0.02 (-0.01;0.06) | 0.07 (0;0.13) | 0.01 (-0.01;0.04) | 0.03 (-0.02;0.08) | 0.15 (0.06;0.24) | 0.08 (0.01;0.14) | 0.04 (-0.01;0.1) | 0.17 (0.07;0.28) | 0.02 (0;0.04) | 0.11 (0.03;0.19) | 0.14 (0.05;0.22) | 0.16 (0.07;0.24) | 0.21 (0.11;0.31) | 0.17 (0.09;0.26) | 0.1 (0.03;0.17) | 0.11 (0.04;0.18) | 0.1 (0.03;0.16) | 0.18 (0.09;0.26) | 0.12 (0.05;0.19) | 0.03 (0;0.07) | 0.09 (0.03;0.15) | 0.11 (0.04;0.18) | 0.14 (0.06;0.22) | 0.16 (0.07;0.24) |
| Foodborne trematodiases | 0.29 (0.16;0.43) | 0.12 (0.02;0.23) | 0.15 (0.06;0.25) | 0.09 (0.01;0.17) | 0.15 (0.06;0.23) | 0.09 (0.02;0.16) | 0.03 (0;0.07) | 0.05 (-0.01;0.1) | 0.03 (0;0.07) | 0.1 (0.02;0.18) | 0.09 (0.02;0.16) | 0.07 (0.01;0.13) | 0.1 (0.03;0.17) | 0.05 (0;0.1) | 0.03 (0.01;0.06) | 0.05 (0;0.09) | 0.02 (-0.01;0.06) | 0.04 (0;0.07) | 0.01 (-0.01;0.03) | 0.02 (-0.01;0.06) | 0 (0;0) | 0.08 (0.02;0.15) | 0.04 (0;0.08) | 0.08 (0.02;0.13) | 0.03 (0;0.07) |
| Rabies | 0.1 (0.03;0.18) | 0.21 (0.09;0.33) | 0.12 (0.04;0.2) | 0.07 (0;0.14) | 0.07 (-0.01;0.14) | 0.06 (0;0.11) | 0 (0;0) | 0.07 (0.01;0.13) | 0.02 (-0.01;0.05) | 0.03 (-0.01;0.06) | 0.06 (0;0.11) | 0.21 (0.11;0.31) | 0.01 (-0.01;0.04) | 0.06 (0.01;0.11) | 0.04 (-0.01;0.08) | 0 (0;0) | 0 (0;0) | 0 (0;0) | 0.01 (-0.01;0.03) | 0 (0;0) | 0 (0;0) | 0 (0;0) | 0 (0;0) | 0.01 (-0.01;0.04) | 0 (0;0) |
| Onchocerciasis | 0 (0;0) | 0 (0;0) | 0 (0;0) | 0.01 (-0.01;0.03) | 0.03 (-0.01;0.07) | 0 (0;0) | 0 (0;0) | 0 (0;0) | 0 (0;0) | 0 (0;0) | 0.01 (-0.01;0.04) | 0.01 (-0.01;0.03) | 0.01 (-0.01;0.03) | 0 (0;0) | 0.02 (-0.02;0.05) | 0 (0;0) | 0 (0;0) | 0 (0;0) | 0.01 (-0.01;0.03) | 0 (0;0) | 0 (0;0) | 0 (0;0) | 0 (0;0) | 0 (0;0) | 0 (0;0) |
