# Supplementary material for: Spatiotemporal trends of neglected tropical disease hospitalizations in Ecuador over 25-years from 2000 to 2024
Source: PLoS Negl Trop Dis. 2026 May 18;20(5):e0013688. doi: 10.1371/journal.pntd.0013688 (PMC13197067; doi:10.1371/journal.pntd.0013688)
Supplement: S3 Table — (DOCX) [file pntd.0013688.s003.docx]

S3 Table. Frequency of hospital discharges for 7 non-endemic NTDs with corresponding hospitalizations reported in Ecuador between 2000 and 2024.

| Non-endemic -NTDs | Year of hospital discharge | | | | | | | | | | | | | | | | | | | | | | | | | Total |
| --- | --- | --- | --- | --- | --- | --- | --- | --- | --- | --- | --- | --- | --- | --- | --- | --- | --- | --- | --- | --- | --- | --- | --- | --- | --- | --- |
|  | 2000 | 2001 | 2002 | 2003 | 2004 | 2005 | 2006 | 2007 | 2008 | 2009 | 2010 | 2011 | 2012 | 2013 | 2014 | 2015 | 2016 | 2017 | 2018 | 2019 | 2020 | 2021 | 2022 | 2023 | 2024 |  |
| Schistosomiasis^1^ | 2 | 1 | 1 | 3 | 2 | 10 | 28 | 70 | 105 | 209 | 35 | 95 | 113 | 86 | 81 | 19 | 16 | 42 | 34 | 22 | 7 | 21 | 19 | 12 | 7 | 1040 |
| Lymphatic filariasis^1^ | 0 | 2 | 0 | 0 | 0 | 0 | 1 | 0 | 3 | 3 | 4 | 0 | 3 | 1 | 5 | 6 | 1 | 1 | 3 | 3 | 1 | 2 | 5 | 1 | 2 | 47 |
| Buruli ulcer^1^ | 0 | 0 | 1 | 0 | 0 | 0 | 1 | 0 | 1 | 0 | 1 | 3 | 1 | 3 | 5 | 3 | 3 | 2 | 2 | 4 | 1 | 2 | 3 | 0 | 6 | 42 |
| Noma | 0 | 1 | 1 | 2 | 4 | 1 | 1 | 1 | 0 | 1 | 1 | 1 | 3 | 0 | 4 | 0 | 1 | 3 | 3 | 5 | 0 | 2 | 3 | 3 | 1 | 42 |
| Trachoma^2^ | 2 | 1 | 0 | 2 | 0 | 1 | 0 | 0 | 0 | 0 | 0 | 1 | 0 | 1 | 1 | 0 | 2 | 0 | 0 | 0 | 0 | 2 | 1 | 0 | 0 | 14 |
| Human African trypanosomiasis^1^ | 0 | 3 | 2 | 2 | 1 | 1 | 1 | 2 | 0 | 0 | 1 | 0 | 0 | 0 | 0 | 0 | 0 | 0 | 0 | 0 | 0 | 0 | 0 | 0 | 1 | 14 |
| Dracunculiasis^2^ | 1 | 0 | 1 | 0 | 0 | 0 | 1 | 0 | 0 | 0 | 1 | 1 | 0 | 0 | 1 | 1 | 0 | 0 | 0 | 1 | 0 | 0 | 0 | 0 | 0 | 8 |

1. No documented history of autochthonous cases reported but present elsewhere in Latin America

2. Cases reported historically but not thought to be transmitted presently
