# Supplementary material for: Spatiotemporal trends of neglected tropical disease hospitalizations in Ecuador over 25-years from 2000 to 2024
Source: PLoS Negl Trop Dis. 2026 May 18;20(5):e0013688. doi: 10.1371/journal.pntd.0013688 (PMC13197067; doi:10.1371/journal.pntd.0013688)
Supplement: S4 Table — (DOCX) [file pntd.0013688.s004.docx]

S4 Table. Frequencies and origins (area of usual residence) of patients hospitalized with non-endemic NTDs in Ecuador between 2015 and 2024 for which data are available.

| Non-endemic NTDs | Ecuador | Elsewhere in Latin America | Outside Latin America |
| --- | --- | --- | --- |
| Dracunculiasis | 2 | 0 | 0 |
| Schistosomiasis | 198 | Colombia - 1 | 0 |
| Lymphatic filariasis | 24 | 0 | Cameroon -1 |
| Trachoma | 4 | Colombia - 1 | 0 |
| Buruli ulcer | 25 | Venezuela - 1 | 0 |
| Noma | 21 | 0 | 0 |
| Human African trypanosomiasis | 1 | 0 | 0 |
