# Supplementary material for: Spatiotemporal trends of neglected tropical disease hospitalizations in Ecuador over 25-years from 2000 to 2024
Source: PLoS Negl Trop Dis. 2026 May 18;20(5):e0013688. doi: 10.1371/journal.pntd.0013688 (PMC13197067; doi:10.1371/journal.pntd.0013688)
Supplement: S5 Table — (DOCX) [file pntd.0013688.s005.docx]

S5 Table. Standardized morbidity ratio (and 95% confidence intervals) of hospitalization rates attributed to 5 most frequent endemic neglected tropical diseases in Ecuador by geoclimatic region and province in census years of 2001, 2010, and 2022.

| Region/Province | Standardized morbidity ratio (95% CI) | | | | | | | | | | | | | | |
| --- | --- | --- | --- | --- | --- | --- | --- | --- | --- | --- | --- | --- | --- | --- | --- |
|  | Dengue and chikungunya | | | Snakebite envenoming | | | Soil-transmitted helminthiases | | | Taeniasis and cysticercosis | | | Scabies and other ectoparasitoses | | |
|  | 2001 | 2010 | 2022 | 2001 | 2010 | 2022 | 2001 | 2010 | 2022 | 2001 | 2010 | 2022 | 2001 | 2010 | 2022 |
| **Coast** |  | | | | | | | | | | | | | | |
| Esmeraldas | 0.94 (0.73-1.22) | 0.34 (0.28-0.42) | 1.87 (1.68-2.09) | 1.18 (0.89-1.57) | 2.87 (2.48-3.32) | 3.89 (3.24-4.68) | 0.82 (0.54-1.25) | 0.23 (0.09-0.54) | 0.93 (0.55-1.57) | 0.12 (0.03-0.5) | 0 (0.00-0.00) | 0 (0.00-0.00) | 0.95 (0.4-2.29) | 1.89 (0.79-4.54) | 2.91 (1.95-4.34) |
| Santo Domingo de los Tsáchilas | 2.6 (2.17-3.11) | 0.73 (0.63-0.86) | 2.55 (2.3-2.83) | 2.1 (1.65-2.69) | 2.01 (1.67-2.44) | 1.69 (1.26-2.27) | 1.73 (1.24-2.42) | 0.68 (0.39-1.2) | 2.31 (1.59-3.34) | 0.25 (0.08-0.77) | 0.36 (0.11-1.1) | 1.58 (0.59-4.2) | 0.27 (0.04-1.89) | 0.92 (0.23-3.69) | 2.06 (1.22-3.48) |
| Manabí | 1.48 (1.32-1.66) | 2.05 (1.94-2.16) | 2.02 (1.89-2.15) | 0.8 (0.66-0.97) | 1.54 (1.37-1.73) | 0.78 (0.62-0.99) | 0.92 (0.74-1.16) | 2.14 (1.79-2.55) | 0.4 (0.24-0.66) | 0.06 (0.02-0.18) | 0.27 (0.13-0.54) | 0.23 (0.06-0.93) | 0.9 (0.53-1.51) | 1.07 (0.54-2.14) | 1.93 (1.43-2.61) |
| Los Ríos | 1.82 (1.58-2.1) | 1.64 (1.52-1.77) | 1.49 (1.35-1.65) | 4.17 (3.72-4.68) | 1.52 (1.3-1.78) | 1.26 (0.98-1.61) | 0.57 (0.38-0.84) | 1.01 (0.72-1.42) | 0.89 (0.57-1.38) | 0.04 (0-0.25) | 0.06 (0.01-0.43) | 0 (0.00-0.00) | 0.72 (0.32-1.6) | 0.72 (0.23-2.22) | 0.71 (0.37-1.36) |
| Guayas | 1.7 (1.59-1.82) | 1.46 (1.4-1.52) | 0.99 (0.93-1.05) | 0.67 (0.59-0.76) | 0.38 (0.33-0.44) | 0.27 (0.21-0.34) | 0.85 (0.74-0.99) | 0.81 (0.68-0.98) | 0.57 (0.44-0.73) | 0.32 (0.24-0.43) | 0.4 (0.28-0.57) | 0.47 (0.26-0.85) | 1.31 (0.98-1.73) | 1.78 (1.27-2.49) | 1.46 (1.18-1.8) |
| Santa Elena | 1.47 (1.13-1.91) | 0.84 (0.71-1) | 3.05 (2.74-3.39) | 0.39 (0.21-0.73) | 0.38 (0.23-0.63) | 0.1 (0.02-0.38) | 0.25 (0.09-0.66) | 0.38 (0.16-0.9) | 0 (0.00-0.00) | 0.29 (0.09-0.89) | 0.47 (0.15-1.45) | 1.04 (0.26-4.15) | 0.33 (0.05-2.32) | 0 (0.00-0.00) | 1.11 (0.5-2.47) |
| El Oro | 2.66 (2.33-3.04) | 3.07 (2.87-3.28) | 1.95 (1.76-2.16) | 1.25 (0.99-1.58) | 0.97 (0.77-1.21) | 0.87 (0.62-1.22) | 1.74 (1.35-2.23) | 0.79 (0.51-1.24) | 0.95 (0.58-1.56) | 0.68 (0.42-1.12) | 0.69 (0.36-1.32) | 0.52 (0.13-2.06) | 0.15 (0.02-1.09) | 0.94 (0.3-2.92) | 1.24 (0.7-2.18) |
| **Andean** |  | | | | | | | | | | | | | | |
| Carchi | 0.04 (0.01-0.3) | 0.03 (0.01-0.1) | 0.02 (0-0.15) | 0 (0.00-0.00) | 0.14 (0.05-0.44) | 0 (0.00-0.00) | 0.67 (0.32-1.41) | 0 (0.00-0.00) | 0.26 (0.04-1.86) | 1.63 (0.9-2.94) | 0.27 (0.04-1.95) | 0 (0.00-0.00) | 1.03 (0.26-4.12) | 0 (0.00-0.00) | 0 (0.00-0.00) |
| Imbabura | 0.07 (0.03-0.19) | 0.03 (0.01-0.06) | 0.03 (0.01-0.08) | 0 (0.00-0.00) | 0.15 (0.08-0.31) | 0.2 (0.08-0.47) | 0.21 (0.09-0.5) | 0.18 (0.06-0.55) | 1.62 (1.01-2.6) | 2.99 (2.23-4) | 1.49 (0.86-2.57) | 1.53 (0.58-4.08) | 1.32 (0.59-2.93) | 0.91 (0.23-3.64) | 0 (0.00-0.00) |
| Pichincha | 0.13 (0.09-0.17) | 0.07 (0.05-0.08) | 0.06 (0.05-0.08) | 0.05 (0.03-0.09) | 0.12 (0.09-0.17) | 0.08 (0.05-0.14) | 0.24 (0.17-0.33) | 0.23 (0.15-0.34) | 0.17 (0.09-0.3) | 1.8 (1.55-2.09) | 1.33 (1.06-1.66) | 0.57 (0.31-1.07) | 0.6 (0.36-1) | 0.37 (0.16-0.9) | 0.1 (0.04-0.28) |
| Cotopaxi | 0.09 (0.04-0.22) | 0.31 (0.24-0.4) | 0.19 (0.13-0.28) | 0.19 (0.09-0.41) | 0.15 (0.08-0.31) | 0.35 (0.18-0.68) | 1.11 (0.76-1.61) | 0.91 (0.56-1.49) | 2.69 (1.88-3.85) | 0.54 (0.27-1.09) | 0.46 (0.17-1.23) | 0 (0.00-0.00) | 1.9 (0.99-3.66) | 1.34 (0.43-4.16) | 0.94 (0.42-2.09) |
| Tungurahua | 0.03 (0.01-0.12) | 0.05 (0.03-0.08) | 0.05 (0.02-0.1) | 0.09 (0.03-0.23) | 0.09 (0.04-0.2) | 0.03 (0-0.23) | 0.87 (0.59-1.28) | 0.75 (0.45-1.25) | 0.56 (0.27-1.17) | 0.71 (0.42-1.2) | 1.32 (0.8-2.19) | 0.94 (0.3-2.91) | 0.37 (0.09-1.46) | 0 (0.00-0.00) | 0.41 (0.13-1.26) |
| Bolívar | 0.19 (0.08-0.45) | 0.23 (0.15-0.35) | 0.23 (0.14-0.4) | 0.34 (0.15-0.77) | 0.85 (0.55-1.31) | 0.93 (0.5-1.72) | 1.27 (0.76-2.1) | 2.16 (1.34-3.47) | 2.59 (1.47-4.55) | 0.98 (0.47-2.06) | 0.25 (0.04-1.79) | 0 (0.00-0.00) | 0.87 (0.22-3.47) | 0 (0.00-0.00) | 0 (0.00-0.00) |
| Chimborazo | 0.08 (0.03-0.19) | 0.08 (0.05-0.12) | 0.06 (0.03-0.12) | 0.07 (0.02-0.23) | 0.07 (0.03-0.18) | 0.16 (0.06-0.42) | 1.02 (0.71-1.47) | 0.57 (0.32-1.04) | 1.49 (0.91-2.43) | 1.35 (0.9-2.03) | 2.01 (1.29-3.11) | 3.4 (1.77-6.53) | 1.1 (0.49-2.45) | 1.19 (0.38-3.7) | 0.32 (0.08-1.27) |
| Cañar | 0.06 (0.01-0.24) | 1.4 (1.2-1.63) | 0.42 (0.29-0.62) | 0.28 (0.13-0.63) | 0.61 (0.38-0.97) | 0.17 (0.04-0.67) | 2.52 (1.83-3.46) | 1.33 (0.77-2.29) | 1.08 (0.48-2.4) | 3.22 (2.22-4.66) | 2.03 (1.09-3.76) | 7.17 (3.73-13.79) | 0.68 (0.17-2.73) | 0 (0.00-0.00) | 0.62 (0.15-2.48) |
| Azuay | 0.05 (0.02-0.13) | 0.08 (0.05-0.11) | 0.06 (0.04-0.11) | 0.11 (0.05-0.24) | 0.15 (0.09-0.26) | 0.09 (0.03-0.25) | 2 (1.61-2.47) | 0.62 (0.39-0.98) | 0.88 (0.54-1.43) | 2.96 (2.36-3.7) | 1.87 (1.3-2.69) | 2.47 (1.37-4.46) | 0.77 (0.35-1.71) | 0.26 (0.04-1.87) | 0 (0.00-0.00) |
| Loja | 0.05 (0.02-0.15) | 0.2 (0.15-0.27) | 0.36 (0.27-0.48) | 0.29 (0.16-0.51) | 1.08 (0.85-1.39) | 0.72 (0.46-1.13) | 0.96 (0.66-1.39) | 1.39 (0.95-2.05) | 1.62 (1.02-2.58) | 3.76 (2.94-4.81) | 7.33 (5.82-9.23) | 5.39 (3.25-8.95) | 1.09 (0.49-2.44) | 0.4 (0.06-2.84) | 0.45 (0.15-1.41) |
| **Amazon** |  | | | | | | | | | | | | | | |
| Sucumbíos | 1.96 (1.44-2.66) | 1.34 (1.12-1.6) | 1.11 (0.87-1.41) | 3.49 (2.63-4.61) | 3.96 (3.22-4.87) | 4.37 (3.28-5.81) | 0.45 (0.17-1.21) | 0.64 (0.27-1.54) | 1.35 (0.64-2.83) | 0.37 (0.09-1.48) | 0.57 (0.14-2.28) | 0 (0.00-0.00) | 4.58 (2.29-9.17) | 0 (0.00-0.00) | 1.06 (0.34-3.29) |
| Napo | 0.31 (0.12-0.83) | 2.48 (2.09-2.94) | 0.76 (0.54-1.08) | 7.48 (5.82-9.62) | 2.97 (2.17-4.07) | 4.42 (3.11-6.28) | 2.64 (1.59-4.39) | 0.21 (0.03-1.47) | 3.33 (1.89-5.87) | 0.33 (0.05-2.32) | 1.01 (0.25-4.03) | 3.28 (0.82-13.12) | 0.86 (0.12-6.11) | 0 (0.00-0.00) | 1.55 (0.5-4.79) |
| Orellana | 0.63 (0.33-1.22) | 2.26 (1.93-2.63) | 1.26 (1-1.59) | 6.18 (4.77-8.01) | 5.51 (4.51-6.74) | 7.72 (6.16-9.68) | 0.49 (0.16-1.53) | 0.63 (0.24-1.68) | 1.16 (0.52-2.58) | 0.88 (0.28-2.72) | 0 (0.00-0.00) | 0 (0.00-0.00) | 2.4 (0.78-7.45) | 0 (0.00-0.00) | 0.72 (0.18-2.89) |
| Pastaza | 0.2 (0.05-0.8) | 2.39 (1.97-2.9) | 1.66 (1.28-2.15) | 17.47 (14.53-21) | 11.38 (9.53-13.6) | 10.85 (8.49-13.86) | 6.68 (4.64-9.61) | 10.37 (7.61-14.14) | 6.29 (4.06-9.75) | 0 (0.00-0.00) | 0 (0.00-0.00) | 1.91 (0.27-13.54) | 5.73 (2.38-13.76) | 0 (0.00-0.00) | 1.16 (0.29-4.63) |
| Morona Santiago | 0.11 (0.03-0.43) | 1.78 (1.5-2.1) | 3.86 (3.4-4.38) | 6.13 (4.86-7.73) | 14.37 (12.74-16.21) | 19.23 (16.7-22.16) | 6.27 (4.79-8.21) | 7.84 (6.05-10.16) | 8.1 (6.11-10.75) | 0.46 (0.12-1.85) | 0 (0.00-0.00) | 2.37 (0.59-9.46) | 1.71 (0.55-5.29) | 4.91 (1.84-13.1) | 0.95 (0.31-2.94) |
| Zamora Chinchipe | 0.24 (0.08-0.75) | 0.72 (0.51-1) | 3.98 (3.35-4.72) | 6.47 (4.92-8.51) | 6.82 (5.48-8.5) | 8.85 (6.76-11.59) | 3.27 (2.06-5.19) | 5.53 (3.68-8.33) | 5.54 (3.39-9.04) | 1.37 (0.51-3.65) | 6.15 (3.4-11.1) | 7.27 (2.73-19.37) | 0.87 (0.12-6.2) | 6.03 (1.95-18.71) | 0.63 (0.09-4.44) |
| **Insular** |  | | | | | | | | | | | | | | |
| Galápagos | 1 (0.32-3.11) | 6.06 (4.81-7.64) | 0.78 (0.35-1.74) | 0 (0.00-0.00) | 0 (0.00-0.00) | 0 (0.00-0.00) | 2.52 (0.81-7.81) | 4.19 (1.57-11.17) | 4.72 (1.52-14.64) | 0 (0.00-0.00) | 0 (0.00-0.00) | 0 (0.00-0.00) | 0 (0.00-0.00) | 0 (0.00-0.00) | 2.8 (0.39-19.89) |
