# Supplementary figures and images for: Spatiotemporal trends of neglected tropical disease hospitalizations in Ecuador over 25-years from 2000 to 2024
Source: PLoS Negl Trop Dis. 2026 May 18;20(5):e0013688. doi: 10.1371/journal.pntd.0013688 (PMC13197067; doi:10.1371/journal.pntd.0013688)

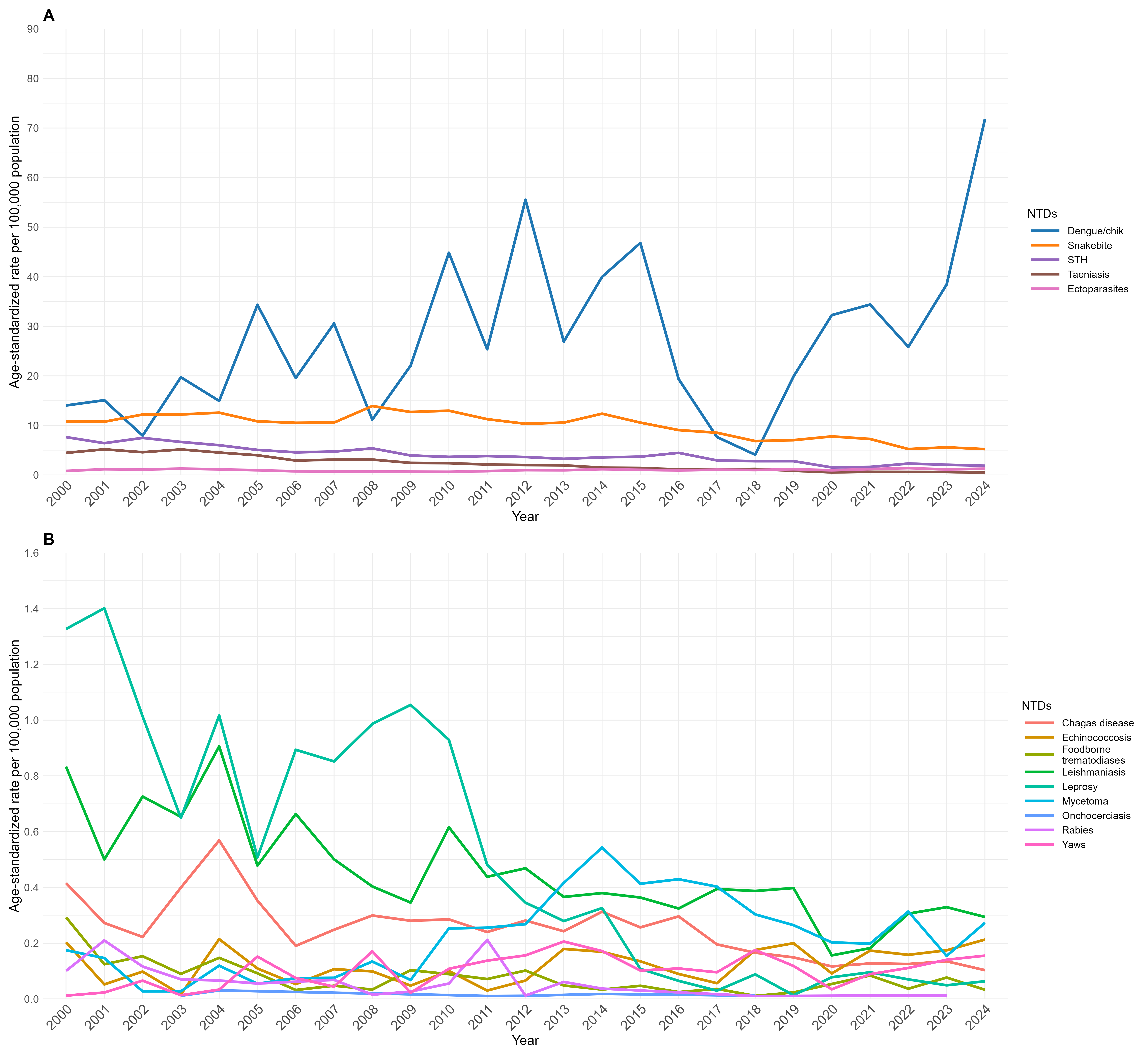

Supplement: S1 Fig — (TIFF) [file pntd.0013688.s006.tiff]

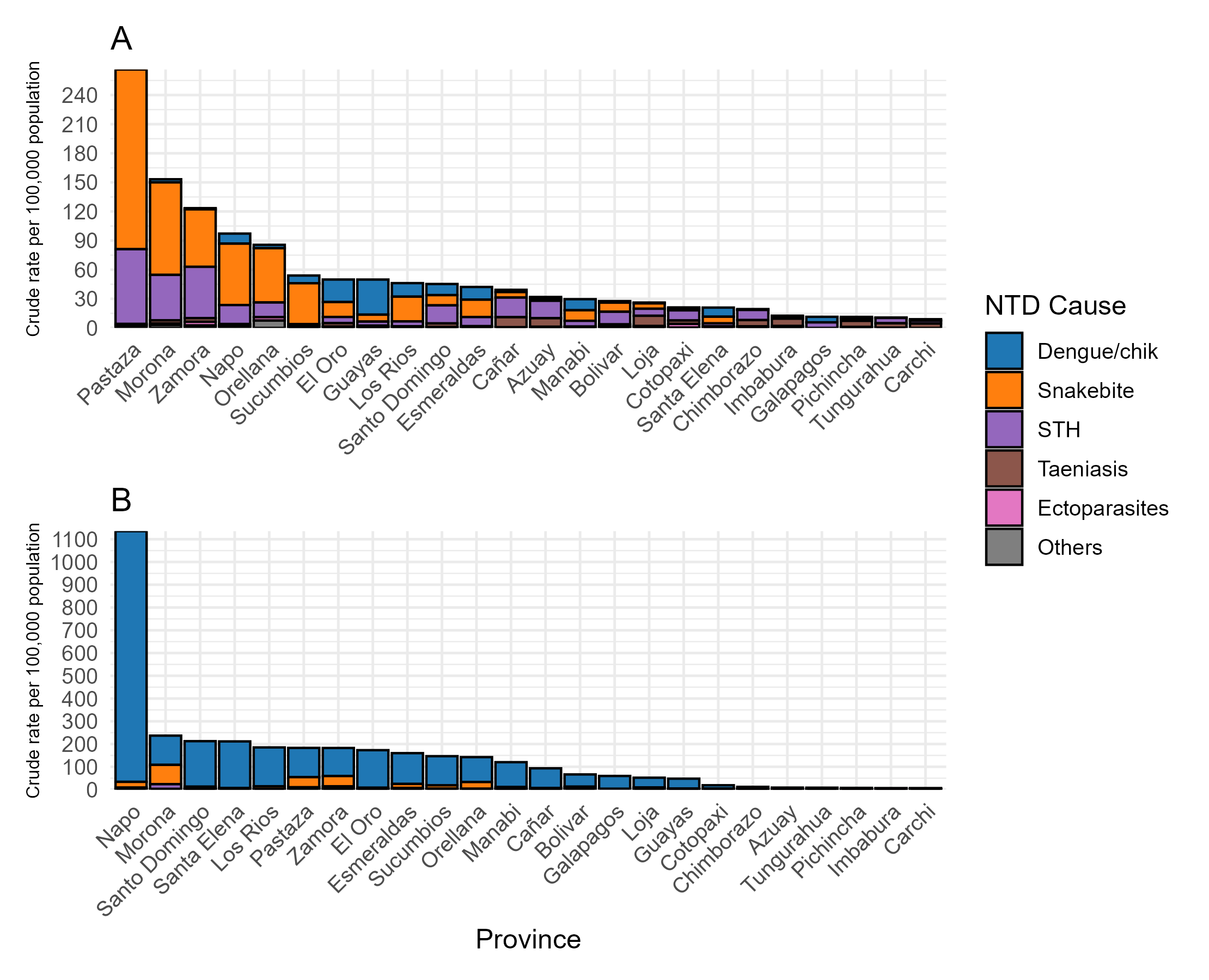

Supplement: S2 Fig — (TIFF) [file pntd.0013688.s007.tiff]
